# Systematic analysis of hippo pathway signaling identifies TEAD1 as a transcriptional regulator of neuroendocrine prostate cancer

**DOI:** 10.1016/j.neo.2026.101321

**Published:** 2026-05-30

**Authors:** Lisha G. Brown, Ilsa M. Coleman, Tony L.H. Chu, Erolcan Sayar, Radhika A. Patel, Brian Hanratty, Mohamed Adil, Dapei Li, Yongtao Li, Holly M. Nguyen, Conner J. Sessions, Erin L. Sweeney, Joshi J. Alumkal, Rui M. Gil da Costa, Yuzhuo Wang, Daniel W. Lin, Lawrence D. True, Ruth Dumpit, Eva Corey, John K. Lee, Peter S. Nelson, Li Xin, Michael C. Haffner, Colm Morrissey

**Affiliations:** a Department of Urology, University of Washington, Seattle, WA, USA; b Division of Human Biology, Fred Hutchinson Cancer Center, Seattle, WA, USA; c Department of Internal Medicine, Rogel Cancer Center, University of Michigan, Ann Arbor, MI, USA; d Department of Laboratory Medicine and Pathology, University of Washington, Seattle, WA, USA; e Department of Urologic Sciences, University of British Columbia, Vancouver, BC, Canada; f Division of Clinical Research, Fred Hutchinson Cancer Center, Seattle, WA, USA; g Department of Medicine, Division of Medical Oncology, University of Washington, Seattle, WA, USA; h Division of Hematology/Oncology, Department of Medicine University of California Los Angeles Jonsson Comprehensive Cancer Center, University of California, Los Angeles, CA, USA

**Keywords:** Prostate, Neuroendocrine, YAP, TEAD1, RBFOX2

## Abstract

Treatment-induced neuroendocrine prostate cancer (NEPC) represents an aggressive form of castration-resistant prostate cancer (CRPC) associated with lineage plasticity and therapeutic resistance. In this study, we investigated the role of the Hippo signaling axis in the transdifferentiation from androgen receptor–positive prostate cancer (ARPC) to NEPC.

RNA sequencing analyses of CRPC metastases revealed coordinated alterations in Hippo pathway components, with decreased expression of YAP1, LATS2, and TEAD2 and increased expression of LATS1, TEAD1, and the RNA splicing regulator RBFOX2 in NEPC. These transcriptional alterations were consistently observed across multiple model systems and patient samples. Epigenetic analyses demonstrated that reduced expression of YAP1, TEAD2, and LATS2 was associated with increased DNA methylation, whereas elevated TEAD1 expression correlated with DNA hypomethylation in NEPC. NEPC selectively retained TEAD1 expression, including a spliced isoform not detected in ARPC. Proteomic interactome analyses revealed that TEAD1 associated with RNA splicing factors and DNA repair proteins. Functional studies showed that TEAD1 knockdown led to the reversion of gene programs associated with epithelial differentiation.

These findings indicate that the conversion of ARPC to NEPC involves coordinated loss of AR, YAP1, and REST activity alongside sustained TEAD1 expression and altered RNA processing. Our data identify TEAD1 as a transcriptional regulator associated with the NEPC state and suggest a role for TEAD1-linked transcriptional and post-transcriptional mechanisms in prostate cancer lineage plasticity.

## Introduction

Androgen deprivation therapy is an effective treatment for metastatic prostate cancer. However, androgen receptor (AR) pathway suppression eventually fails leading to castration-resistant prostate cancer (CRPC). Resistance mechanisms associated with metastatic CRPC include conversion to AR-null phenotypes, such as neuroendocrine prostate cancer (NEPC) [1]. Transdifferentiation of ARPC to NEPC is accompanied by, or driven by the loss of the AR expression, inactivation of the repressor element silencing transcript factor (REST), and the expression of ASCL1 or similar master regulators of neuroendocrine differentiation [[1], [2], [3], [4], [5]]. Another significant alteration in NEPC that has previously been described is the silencing of the transcription factor YAP, a key component of the Hippo signaling pathway [6]. However, a detailed analysis of Hippo pathway activity in CRPC is currently lacking.

Broadly, the Hippo pathway is a multicomponent signaling program that regulates tissue homeostasis, organ size, and regeneration [7]. YAP is a Hippo pathway component that functions as a transcriptional co-activator shown to promote cancer initiation, progression, invasion, and therapy resistance across different tumor types [8]. YAP exerts its activities mostly via interactions with Transcriptional Enhanced Associated Domain (TEAD) transcription factors [8,9]. When the Hippo pathway is switched ‘off’, YAP enters the nucleus and interacts with the TEAD family of transcription factors promoting proliferation [7]. In contrast, LATS (LATS1/2) proteins suppress the activity of YAP [10]. In epithelial cells, when the Hippo pathway is switched ‘on’, YAP (and its paralogue TAZ) is phosphorylated by the LATS proteins targeted for degradation, blocking proliferation and migration. In most epithelial solid tumors, YAP promotes proliferation. As such, YAP is expressed in the majority of AR-positive CRPC metastases. However, despite high proliferation rates, prior studies have determined that in NEPC, YAP expression is lost [6,11]. To more fully evaluate the role of Hippo-pathway signaling in metastatic PC, we evaluated Hippo signaling components by assays of mRNA levels, protein abundance, and splicing events in AR-positive CRPC (ARPC) and NEPC. Our results identified TEAD1 as a potential transcriptional master regulator in NEPC. In addition, we observed that RBFOX2 a pre-RNA splicing regulator that promotes the inclusion of exon 6 in TEAD1 mRNAs [12] was associated with increased TEAD1 splicing in NEPC. Furthermore, we determined the TEAD1 interactome correlated with the spliceosome in NEPC cells, and demonstrated that TEAD1 knockdown *in vitro* suppressed the expression of epithelial genes in NEPC. These data indicate that TEAD1 plays a crucial role in maintaining the NEPC phenotype in CRPC.

## Materials and methods

### Tissue acquisition

Tissue Acquisition Necropsy (TAN) tumor samples were obtained from patients who died from CRPC under the aegis of the Prostate Cancer Donor Program at the University of Washington (UW) (IRB protocol # 2341). All animal experiments were approved by the UW Institutional Animal Care and Use Committee and according to NIH guidelines (IACUC protocol # 3202-01). Male mice (CB‐17 SCID, Charles River Laboratory) were implanted subcutaneously with tumor bits and tumors were passaged in animals. Tumor phenotype was determined by RNAseq and verified by immunohistochemistry (IHC) using antibodies to synaptophysin and AR (**Supplemental Table S1**).

### Cell lines

LNCaP (ATCC; CRL-1740), C4-2B (Obtained from L Chung), DU145 (ATCC; HTB-81), LAPC4 (ATCC; CRL-13009), VCaP (ATCC; CRL-2876), 293T (ATCC; CRL-3216), 22Rv1 (obtained from S Plymate), LTL331R derived from the LTL331R tumor (obtained from YZ Wang) [13], and MSKCC EF1 cells (obtained from J Lee) [14], were maintained in RPMI-1640 Media (Gibco, Life Technologies) with 10% fetal bovine serum (Atlanta Biologicals). NCI-H660 cells (ATCC; CRL-5813) were maintained in complete HITES media (ATCC formulation).

### Viability and apoptosis assays

Viability was assessed 72 h post-treatment using the CellTiter Glo 3D‐Cell Viability Assay (Promega) according to manufacturer's protocols. The LATS1/2 inhibitor: LATS-IN-1 and TEAD inhibitors: VT103, K-975, and MYF-01-37 were purchased from MedChemExpress.

### Knockdown

7.5 μL of TransIT-siQUEST® (Mirus Bio #2110) were added to 200 μL of MEM media then 6.8 μL at a 10 μM concentration of TEAD1 (Entrez gene 7003) or RBFOX2 (Entrez gene 23543) ON-TARGETplus SMARTPool siRNA (Dharmacon) were added to a final concentration of 5 nM/ well. After 24 h, RNA was isolated using STAT-60 and used for RNAseq.

### Immunoblot analysis

Protein was extracted from cells using the Nuclear Extract Kit (Active Motif) according to manufacturer’s protocol. Quantification of total protein was determined using the RcDc Protein Assay (Bio-Rad Laboratories) according to manufacturer’s protocols. Twenty micrograms of total protein lysate were electrophoresed on 4-15% Bis-Tris gels (Bio-Rad Laboratories) with 1x Tris/Glycine/SDS Buffer (Bio-Rad Laboratories). The proteins were transferred to nitrocellulose membranes that were blocked with 5% Blotting-Grade Blocker (Bio-Rad Laboratories) in TBS/0.1% Tween-20 and subsequently probed with primary and secondary antibodies (**Supplemental Table S1**). Proteins were visualized using Clarity Western ECL Substrate (Bio-Rad Laboratories) on a Chemi-Doc Imaging System (Biorad). For the YAP/TAZ Western blot, cells were lysed in RIPA buffer (20 mM Tris-HCl, pH 7.5, 150 mM NaCl, 1 mM Na_2_EDTA, 1 mM EDTA, 1% NP-40, 1% sodium deoxycholate, 2.5 mM sodium pyrophosphate, 1 mM β-glycerophosphate, 1 mM Na_3_VO_4_) supplemented with protease inhibitors and phosphatase inhibitors (GenDEPOT). The protein concentration was measured using a Bradford Assay kit (BioRad). Western blot analysis was performed with precast gradient gels (4-12%) (GenScript) using standard methods. Proteins were transferred onto 0.2 µm nitrocellulose membrane (Bio-Rad). Membranes were blocked in 5% non-fat milk (Santa Cruz Biotech) in Phosphate Buffered Saline with Tween-20 (PBST), and then incubated with primary antibodies against YAP/TAZ (1:500) (**Supplemental Table S1**) overnight at 4°C, washed in PBST, incubated with an HRP-conjugated secondary antibody (R&D Systems Inc) and developed by the ECL reagent (Thermal Scientific). The bands were visualized by an Amersham Imager 600 (Cytiva).

### Immunoprecipitation

20 × 10^6^ MSKCC EF1 cells were cultured in RPMI-1640 Media (Gibco, Life Technologies) with 10% fetal bovine serum (Atlanta Biologicals). Protein was isolated using a RIPA lysis extraction buffer (Thermo Scientific; 89900), per manufacturer’s guidelines for suspension cultured mammalian cells. Protein concentration was determined by the RC DC protein assay (Bio-Rad; 5000119). Three hundred μg of protein was added to antibody conjugated Dynabeads^TM^ Protein G beads (Invitrogen; 10007D) bound with 5 μg of anti-hnRNP U or 5 μg of mouse IgG as control (**Supplemental Table S1**) and immunoprecipitated per manufacturer’s protocol. Immunoprecipitated samples were assessed by immunoblot analysis using a TEAD1 antibody (**Supplemental Table S1**) as described above.

### RNA sequencing

Sequencing reads were mapped to the GRCh38 human reference genome using STAR v2.7.3a. LuCaP Patient-derived xenograft (PDX) data were also aligned to the mm10 mouse genome. All subsequent analyses were performed in R. LuCaP PDX sequencing reads derived from mouse were removed using XenofilteR. Gene level abundance was quantified using GenomicAlignments and transformed to log_2_ FPKM in edgeR. Differential expression between groups was assessed using limma-voom, filtered for a minimum expression level using the filterByExpr function with default parameters prior to testing, and using the Benjamin-Hochberg false discovery rate (FDR) adjustment.

### Analysis of RNA splicing

RTPCR and RNAseq was used to determine *TEAD1* expression and splicing ‘in’ of a 12bp exon 6 into *TEAD1*. First-strand cDNA synthesis was performed with 1 µg of RNA using an Advantage RT-for-PCR Kit (Clontech Laboratories). PCR was performed using either Platinum SYBR Green qPCR SuperMix-UDG (Invitrogen; qPCR and Screening PCR) or HotStarTaq Plus Master Mix (Qiagen; Sequencing PCR) on a Rotor-Gene Q (Qiagen). *TEAD1* primers were used for RTPCR and visualization on an agarose gel [12]. All primer set sequences are listed in **Supplemental Table S1**. Splice junction RNAseq reads were extracted from STAR-aligned BAM files using the sjFromSAMcollapseUandM.awk script in the STAR package. Spliced reads mapping uniquely to the *TEAD1* E6 12 bp junction for both PDX models and patient specimens were quantified and reported as spliced reads per million.

### Single-nucleus RNA sequencing (snRNAseq)

Single-nucleus suspensions were prepared using the Chromium Nuclei Isolation Kit (10x Genomics) according to the manufacturer’s protocol. Briefly, nuclei were isolated from frozen tissue samples, filtered to remove debris and aggregates, and counted prior to loading. Single nuclei were quantified using hemocytometer for downstream pipeline of single cell/nuclei RNA sequencing. Single-nucleus 3′ gene expression libraries were generated using the Chromium Single Cell 3′ Gene Expression platform (Single Cell A Chip Kit, Single Cell 3′ Library & Gel Bead Kit v2, and i7 Multiplex Kit; 10x Genomics). Approximately 10,000 nuclei per sample were targeted for recovery. Gel bead–in–emulsion (GEM) generation, reverse transcription, cDNA amplification, and library construction were performed following the manufacturer’s instructions. Final libraries were sequenced on an Illumina NextSeq platform (P3 flow cell configuration) to obtain paired-end reads. Sequencing data were processed using Cell Ranger (v6.1.1), and aligning to the GRCh38 human reference genome, and generation of gene-barcode count matrices. Downstream transcriptomic analysis and visualization were performed using Parse Biosciences Trailmaker. Log-normalized gene expression matrices were analyzed to generate dimensionality reduction embeddings, including Uniform Manifold Approximation and Projection (UMAP), for visualization of transcriptional heterogeneity across nuclei.

### Pathway analysis

Genome-wide RNAseq gene expression results were ranked by their limma statistics and used to conduct Gene Set Enrichment Analysis (GSEA) to determine patterns of pathway activity utilizing the curated pathways from within the MSigDB v2023.1.Hs. Analysis was also performed using the enrichr platform (https://maayanlab.cloud/Enrichr/) [[15], [16], [17]] and the Gene Ontology Resource (https://geneontology.org/) [18,19].

### DNA methylation analyses

Whole-genome bisulfite sequencing analyses from LuCaP PDX models were performed as published previously [20]. Briefly, raw whole-genome bisulfite sequencing (WGBS) reads were first trimmed using Trim Galore [0.6.6] and then aligned to the UCSC hg19 reference genome using Bismark [0.23.0] [21]. Bismark was further used to deduplicate the alignments and extract methylation call files to report the percentage of methylated cytosines and the coverage at each position. Additional previously published WGBS data from the SU2C West Coast Dream Team was analyzed [22]. Genome scale methylation analyses of 14 LuCaP PDX DNAs were carried out using Infinium MethylationEPIC BeadChip arrays (Illumina) as described previously [23]. Raw data were analyzed in the minfi package in R and samples were normalized using the subset-quantile within array normalization (SWAN) method [24]. Probes with a detection p value of >0.01 in 50% or more of samples and probes that contained a SNP at the CpG interrogation site or at the single nucleotide extension were removed. DNA methylation patterns were inspected in IGV and methylation levels of the DMR were extracted [25]. Whole-genome bisulfite sequencing and EPIC array data are publicly available on Gene Expression Omnibus (GEO), accession number GSE205056.

### Rapid immunoprecipitation mass spectrometry of endogenous protein (RIME)

To map TEAD1 protein interactions, MSKCC EF1 cells were crosslinked with formaldehyde, lysed, sonicated and subjected to **R**apid **I**mmunoprecipitation **M**ass spectrometry of **E**ndogenous proteins (**RIME**) analyses as described previously [26,27]. TEAD1-associated complexes were precipitated using TEAD1 antibody (Cell Signaling; 12292), digested with trypsin/lysC and resulting peptides were subjected to liquid chromatography mass spectrometry analysis on a Thermo Scientific Orbitrap Eclipse (*Johns Hopkins Proteomics Core Facility*). All samples were run in replicates and resulting spectra were analyzed using the Thermo Scientific Proteome Discoverer and Scaffold software packages.

### Immunohistochemistry

Five-micron sections of tissue microarrays (TMAs) were deparaffinized and rehydrated in sequential xylene and graded ethanol. Antigen retrieval was performed in 10 mM citrate buffer (pH 6.0) in a pressure cooker for 30 minutes. Endogenous peroxidase and avidin/biotin were blocked respectively (Vector Laboratories). Sections were then blocked with 5% normal goat-horse-chicken serum, incubated with primary antibodies (**Supplemental Table S1**), incubated with biotinylated secondary antibody (Vector Laboratories), followed by ABC reagent (Vector Laboratories), and stable DAB (Thermo Fisher Scientific). All sections were lightly counterstained with hematoxylin and mounted with Cytoseal XYL (Richard Allan Scientific). Mouse or rabbit IgG were used as negative controls.

### Statistics

Sample size for each experiment is indicated in the figure legends. Statistical analyses for RNAseq expression were performed as indicated using R software. Statistical comparisons between CRPC molecular phenotypes were conducted using an unpaired t-test using Prism (GraphPad).

### Luciferase reporter assay

For the YAP luciferase reporter assay, a 2 Kb DNA fragment containing firefly luciferase driven by a YAP/TAZ-responsive synthetic promoter (8xGTIIC-Luciferase) was PCR amplified from an 8xGTIIC-Luciferase plasmid (Addgene, #34615) and cloned into the FU-CGW lentiviral plasmid [28]. Lentivirus preparation, titering, and infection of EF1 and 293T cells were performed as described previously [28]. Seventy-two hours later, luciferase activity was measured using the Luciferase Assay Reagent (Promega).

### Data availability

Transcriptome analyses of the UW TAN CRPC: GSE147250, GSE228283, SU2C CRPC: cBioPortal; prad_su2c_2019, LuCaP PDX models: GSE199596, LTL331 PDX: ERP010791, and cell lines: GSE228283, GSE288591, GSE302516 were conducted using previously published RNA sequencing datasets. RNA sequencing data, whole-genome bisulfite sequencing, and EPIC array methylation data generated in this report have been deposited at Gene Expression Omnibus (GEO) and can be accessed through accession number accession numbers GSE205056 and GSE315920. snRNAseq data currently being deposited. Additionally, all primer set sequences and antibodies used in this report are listed in **Supplemental Table S1**.

## Results

### Loss of YAP signaling and rewiring of the TEAD axis in NEPC

Loss of YAP expression in NEPC has been reported previously [6,9,11]. To confirm and contextualize this phenotype in the datasets used for our analyses, we assessed transcript abundance of established NEPC markers across three previously published CRPC cohorts: the University of Washington prostate cancer TAN program, the SU2C International Dream Team cohort, and the LuCaP PDX series (**Supplemental Fig. 1A**). Expression of canonical NEPC and AR-lineage markers, including ASCL1, SOX2, INSM1, SRRM3, ACTL6B, SYP, and AR, clearly distinguished AR-positive prostate cancer (ARPC) from NEPC across all three datasets (**Supplemental Fig. 2**), consistent with prior classifications [2].

To validate tumor phenotype at the protein level, we performed IHC on tissue microarrays containing NEPC (LuCaP 208.2, 208.1, 173.1, 145.2, 145.1, 93, and 49) and ARPC (LuCaP 170.1, 147, 136, 96, 70, 35, and 23.1) xenografts. NEPC tumors exhibited loss of AR with concomitant expression of ASCL1, synaptophysin (SYP), BAF53B (a marker of REST inactivation), and SOX2, confirming their neuroendocrine phenotype (**Supplemental Fig. 3**).

**Fig. 1:**
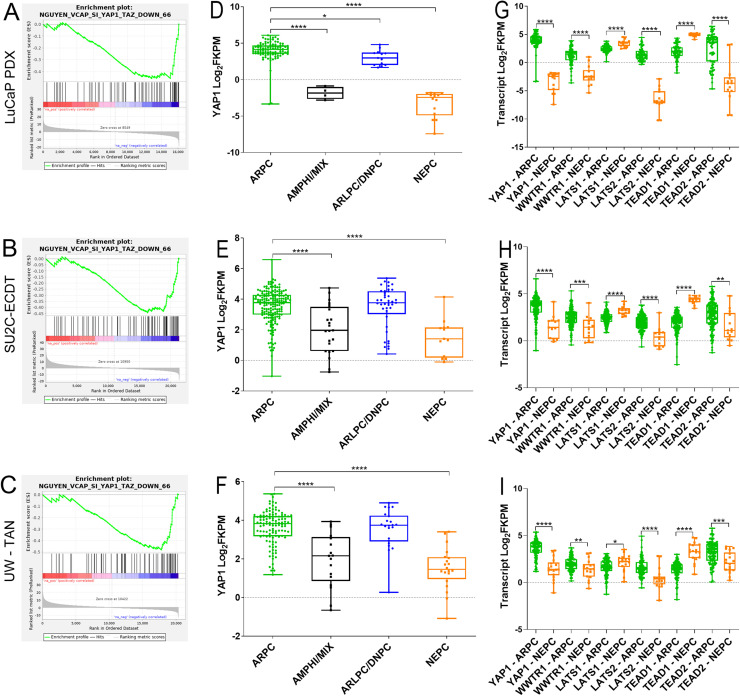
**YAP and YAP Pathway-associated gene expression in ARPC and NEPC**. (A through C) enrichment plots of the Nguyen siYAP/TAZ down-regulated signature comparing ARPC and NEPC LuCaP models (FDR.q.val = 0.0009), SU2C (FDR.q.val = 0.0006), and UW TAN specimens (FDR.q.val = 0.0021). (D through F) YAP1 Transcript abundance in 45 LuCaP PDX models, 270 biopsies from the SU2C dataset, and 132 metastases from 77 patients from the UW TAN program. Androgen receptor positive (ARPC), amphicrine and ARPC/NEPC mixed (AMPHI/MIX), AR-low and double negative (ARLPC/DNPC), and neuroendocrine prostate cancer (NEPC). (G through I) Transcript abundance of YAP-pathway associated genes (*YAP1, WWTR1, LATS1, LATS2, TEAD1*, and *TEAD2*) in (A) 45 LuCaP PDX models, (B) 270 biopsies from the SU2C dataset, and (C) 132 metastases from 77 patients from the UW TAN program. Androgen receptor positive (ARPC; green) and (NEPC; orange). * p<0.05; ** p<0.01; *** p<0.001; **** p<0.0001.

Differential expression analyses using volcano plots and gene set enrichment analysis (GSEA) consistently demonstrated marked transcriptional divergence between ARPC and NEPC. NEPC tumors showed enrichment of pathways associated with proliferation and neuroendocrine differentiation, including E2F_TARGETS, G2M_CHECKPOINT, and PANCREATIC_BETA_CELLS, whereas ARPC tumors retained enrichment of ANDROGEN_RESPONSE and MYC_TARGETS_V1/V2 pathways (**Supplemental Fig. 1B–E**). Notably, across all three CRPC datasets, the NGUYEN_VCAP_SI_YAP1_TAZ_DOWN_66 gene set (genes altered by loss of YAP1/TAZ activity) was significantly negatively enriched in NEPC relative to ARPC [29], confirming suppression of YAP/TAZ signaling in NEPC (Fig. 1A–C).

To directly assess YAP/TAZ activity, we examined YAP and TAZ protein expression in prostate cancer cell lines. The NEPC line MSKCC EF1 was identified as one of the lines with minimal YAP/TAZ expression (**Supplemental Fig. 4A**). Consistent with this finding, a YAP/TAZ-responsive luciferase reporter assay demonstrated negligible transcriptional activity in MSKCC EF1 cells compared with control 293T cells (**Supplemental Fig. 4B**), confirming functional inactivity of the YAP pathway in this NEPC model.

Analysis of YAP1 transcript levels across the LuCaP, SU2C, and UW-TAN datasets further revealed that ARPC, AR-low prostate cancer (ARLPC), and double-negative prostate cancer (DNPC) tumors retained significantly higher YAP1 expression than amphicrine or NEPC tumors (Fig. 1D–F). Importantly, YAP1 was not the only component of the Hippo–YAP pathway altered in NEPC. Across all three datasets, NEPC tumors exhibited coordinated downregulation of YAP1, WWTR1 (TAZ), LATS2, and TEAD2, alongside increased expression of LATS1, TEAD1 (Fig. 1G–I).

We further confirmed the differential expression pattern of Hippo pathway components in multiple model systems, including additional LuCaP PDX models, prostate cancer cell lines, and the LTL331 transdifferentiation model [30] (**Supplemental Fig. 5**). One exception was partial retention of TEAD2 expression in LuCaP 93 and relapsed LTL331R NEPC tumors, where TEAD2 transcript levels were reduced but still detectable, suggesting context-dependent regulation during transdifferentiation.

Next, because bulk RNAseq results can be confounded by stromal, and immune cell contamination, we evaluated Hippo–YAP pathway expression using snRNAseq from two ARPC and two NEPC liver CRPC metastases in the UW-TAN dataset (**Supplemental Fig. 6A through D)**. The prostate epithelial and neuroendocrine aspect was verified using AR and KLK3, and ASCL1 and NRXN1 respectively. These analyses recapitulated the bulk RNAseq findings, confirming reduced YAP signaling and elevated TEAD1 expression in NEPC tumor cells (Fig. 5).

### LATS1 functions independently of YAP in NEPC

Despite reduced LATS2 expression in NEPC, LATS1 expression was maintained. To probe the functional significance of this observation, we treated NEPC cells with the dual LATS1/2 inhibitor LATS-IN-1. In the absence of YAP and LATS2, the expected target of LATS-IN-1 would be LATS1. Treatment with LATS-IN-1 had no inhibitory effects on NEPC cell growth and in some contexts modestly promoted proliferation (**Supplemental Fig. 7**), suggesting that LATS1 may function as a tumor suppressor in YAP-negative NEPC.

### TEAD1 is retained and epigenetically regulated in NEPC

We next examined the expression of all four TEAD transcription factors in CRPC tumors. Among the TEAD family members we observed that TEAD1 was significantly upregulated in NEPC, whereas TEAD2 and TEAD3 were significantly downregulated; TEAD4 expression was low and not altered (**Supplemental Fig. 8**). Immunohistochemical analysis of LuCaP PDX tumors demonstrated nuclear localization of TEAD1 in NEPC tumors, with limited nuclear TEAD1 in ARPC tumors (**Supplemental Fig. 9**).

**Fig. 2:**
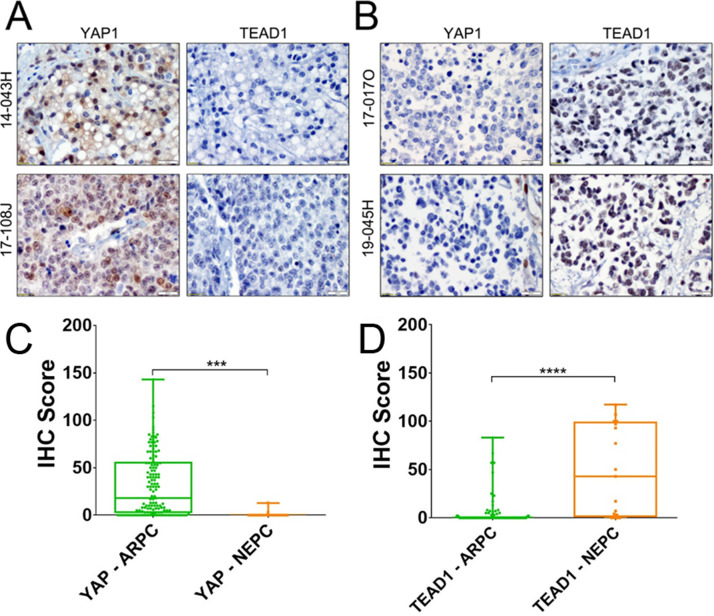
**TEAD1 is present in the nucleus of NEPC cells in patient metastasis.** YAP and TEAD1 expression in (**A**) ARPC and (**B**) NEPC patient specimens. Bar = 20 microns. IHC Scores for (**C**) YAP and (**D**) TEAD1 expression in ARPC versus NEPC. Androgen receptor positive (ARPC; green) and (NEPC; orange). *** p<0.001; **** p<0.0001.

The differential expression patterns of Hippo components in ARPC versus NEPC tumor differentiation was recapitulated in patient metastases. Among 156 metastatic tumors analyzed, 17 were classified as NEPC and 139 as ARPC based on AR, NKX3-1, and synaptophysin staining. YAP and TEAD1 IHC revealed reciprocal expression patterns, with NEPC metastases showing loss of YAP and robust nuclear TEAD1 immunoreactivity, whereas ARPC metastases retained YAP and exhibited low TEAD1 immunoreactivity (Fig. 2A–D).

**Fig. 3:**
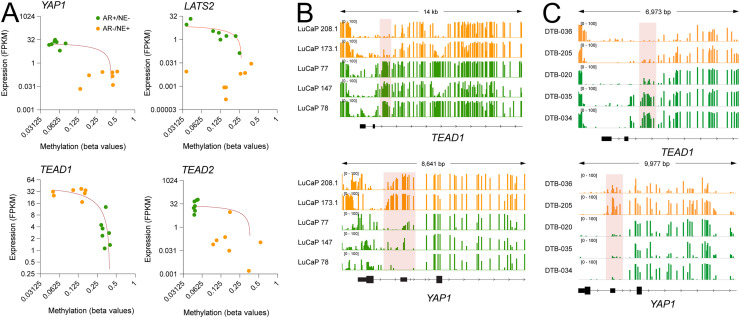
**YAP-pathway gene methylation.** CpG methylation: To determine DNA methylation alteration in YAP-associated genes, whole-genome bisulfite sequencing and EPIC array was used. Methylation data was extracted for relevant differentially methylated regions (DMRs) and correlated with transcript levels determined by RNAseq. Methylation of YAP-associated genes in (A) ARPC (AR+NE-) and NEPC (AR-/NE+) LuCaP PDX models. (B) Examples of whole-genome bisulfite sequencing identifying a differentially methylated region of *TEAD1* that is hypomethylated in NEPC and *YAP1* that is hypermethylated in NEPC LuCaP PDX models and in (C) the SU2C West Coast Dream Team methylation dataset. Androgen receptor positive (ARPC; green) and (NEPC; orange).

Given that treatment-induced transdifferentiation from ARPC to NEPC is primarily epigenetic [[31], [32], [33]], we assessed the DNA methylation status of Hippo–YAP pathway genes. Loss of YAP1, TEAD2, and LATS2 expression correlated with increased promoter methylation in NEPC PDX models, whereas TEAD1 exhibited reduced methylation, consistent with transcriptional activation (Fig. 3A). These methylation changes were validated by whole-genome bisulfite sequencing in LuCaP PDXs and in the SU2C West Coast Dream Team methylation dataset [34] (Fig. 3B,C).

### TEAD1 splicing defines NEPC transcriptional output

**Fig. 4:**
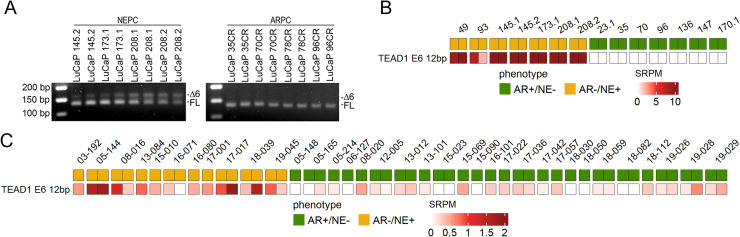
***TEAD1* splicing occurs in NEPC.** (A) RTPCR of *TEAD1* splicing shows the presence of a secondary spliced *TEAD1* product in NEPC samples and an absence in ARPC samples. (B) A heatmap of *TEAD1* splicing in ARPC (AR+, NE-; green) and NEPC (AR-, NE+; orange) LuCaP PDX, and (C) patient metastatic sites as defined by spliced reads per million (SRPM) [12]. (AR+, NE-; green) and NEPC (AR-, NE+; orange).

RBFOX1, RBFOX2, and RBFOX3 are tissue-specific splicing regulators [35]. Across all three CRPC RNAseq datasets, RBFOX1-3 transcripts were significantly increased in NEPC with RBFOX2 expressed at substantially higher levels than RBFOX1 or RBFOX3, indicating RBFOX2 as the dominant family member in NEPC (**Supplemental Fig. 10**). RBFOX2 also regulates alternative splicing of TEAD1, promoting inclusion of exon 6 (TEAD1Δ6), which enhances transcriptional activity of TEAD1, independent of YAP [12]. Analysis of LuCaP PDX models revealed selective expression of TEAD1Δ6 in NEPC (Fig. 4A). RNAseq confirmed consistent expression of TEAD1Δ6 in NEPC PDXs (Fig. 4B), and patient metastases demonstrated increased TEAD1 splicing events in NEPC relative to ARPC (Fig. 4C). Collectively, these data identify TEAD1Δ6 as the predominant TEAD isoform driving TEAD-associated transcription in NEPC.

**Fig. 5:**
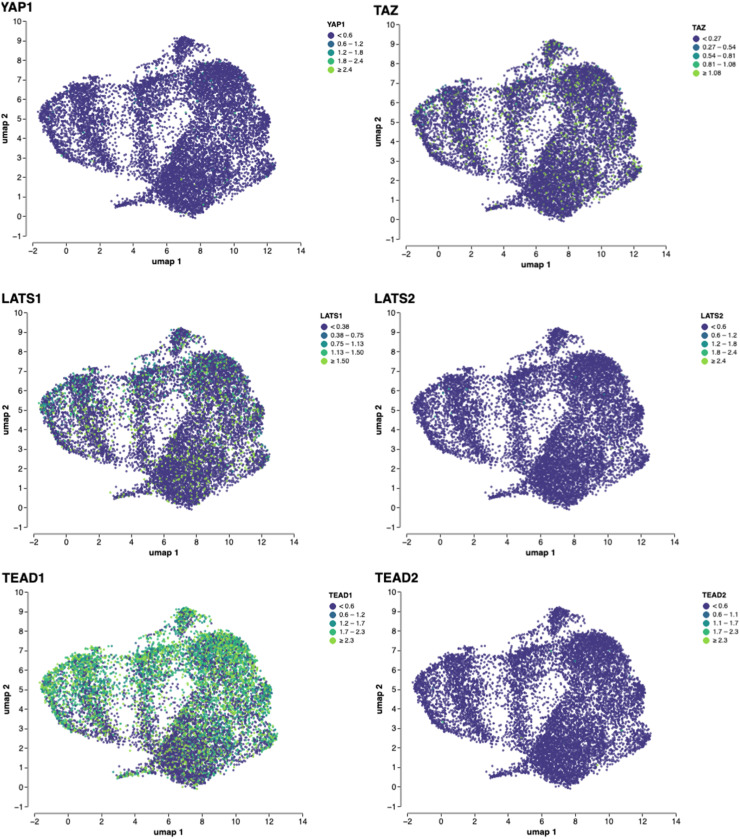
**snRNAseq of an NEPC patient liver metastasis.** tSNE plot of YAP-pathway associated genes from an NEPC liver metastasis from a prostate cancer patient. Gene expression values are log-normalized; values above the 95^th^ percentile were capped for visualization on the UMAP.

To determine if RBFOX2 was directly associated with TEAD1 splicing, we knocked down RBFOX2 in MSKCC EF1 cells, however knockdown did not lead to a decrease in TEAD1 exon 6 splicing. It is possible we do not see a significant difference as the knockdown was not sufficient to impact splicing in the NEPC cells. Additionally, an increase in RBFOX2 itself may not be sufficient to promote splicing and a co-factor may be required.

Knockdown of RBFOX2 in MSKCC EF1 cells did reduce expression of SLC7A11, a regulator of PI3K signaling (**Supplemental Fig. 11A**; [36]) and GSEA further demonstrated suppression of the KEGG PI3K signaling pathway following RBFOX2 knockdown (**Supplemental Fig. 11B**). Given prior evidence linking RBFOX2 to PI3K-dependent tumor growth [37], these findings suggest a similar oncogenic role for RBFOX2 in NEPC.

### TEAD1 regulates epithelial differentiation programs

To delineate the role of TEAD1 on proliferation and survival of NEPC cells, we used pharmacologic inhibition of TEAD using VT103, K-975, and MYF-01-37, agents that disrupt TEAD palmitoylation and/or YAP–TEAD interactions, but they had minimal effects on NEPC cell viability (**Supplemental Fig. 12**). However, these compounds are optimized for YAP-dependent TEAD activity and may not effectively inhibit YAP-independent or TEAD1Δ6-mediated transcription.

**Fig. 6:**
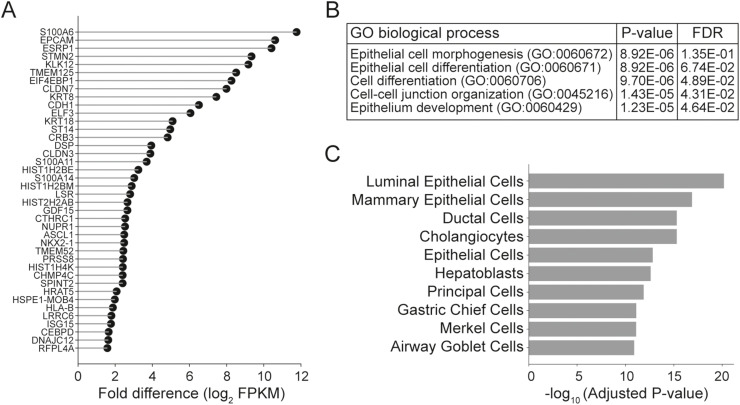
**TEAD1 suppresses epithelial gene expression.** (A) Differential gene expression of protein coding genes following siTEAD1 knockdown in MSKCC EF1 cells with a log_2_FPKM > 0 and a fold difference in expression >2. (B) Gene Ontology Resource PANTHER Overrepresentation Test of the 40 genes in A. (C) Enrichr analysis of the same 40 genes identified in A using the PanglaoDB Augmented 2021 cell type specific dataset.

To directly assess TEAD1 function, we performed TEAD1 knockdown in MSKCC EF1 cells. RNAseq analysis identified the de-repression of 40 epithelial-associated genes (>2-fold change, log2 FPKM > 0) following TEAD1 knockdown (Fig. 6A). Gene Ontology analysis revealed significant enrichment of epithelial morphogenesis and differentiation pathways (Fig. 6B), while Enrichr analysis demonstrated increased expression of luminal epithelial gene programs (Fig. 6C). In contrast, TEAD1 knockdown in NCI-H660 cells did not produce similar transcriptional changes, highlighting context-specific dependency. However, there were a small number of genes that were in common between the MSKCC EF1 and NCI-H660 cells (**Supplemental Fig. 13A**). Hallmark pathway analysis also identified apoptosis and hypoxia pathways along with inflammatory pathways were increased after TEAD1 knockdown. Suggesting cellular stress in response to TEAD1 knockdown. In addition, androgen response genes were up in both cell lines, as would be expected where TEAD1 repressed epithelial genes in the neuroendocrine phenotype, however, the increase is limited (**Supplemental Fig. 13B**). Unlike the de-repression of epithelial-associated genes by TEAD1 knockdown, no substantial decrease in neuroendocrine-associated genes was observed (**Supplemental Fig. 13C**).

### TEAD1 interacts with splicing and RNA-processing machinery in NEPC

Given the absence of YAP in NEPC, we interrogated the TEAD1 interactome using RIME [27] in MSKCC EF1 cells. We identified 428 interacting proteins, including TEAD1, with several interactions validated by immunoprecipitation (**Supplemental Fig. 14A; Supplemental Tables S2–S3**). Enrichr analysis of high-confidence interactors revealed strong enrichment for mRNA splicing proteins via the spliceosome (GO Biological Process 2025; P = 1.02 × 10⁻¹⁸) (**Supplemental Fig. 14B**).

Notably, the TEAD1 interactome substantially overlapped with a previously described SOX2 interactome in glioblastoma [38], including heterogeneous nuclear ribonucleoproteins, RNA-binding proteins, DNA repair factors, and ribosomal proteins (**Supplemental Table S4**). Given that SOX2 is a known driver of lineage plasticity in prostate cancer and NEPC [39,40] and has established roles in alternative splicing, these findings suggest functional convergence between TEAD1 and SOX2 in the context of regulating cell lineage identity.

Importantly, several TEAD1-associated proteins—hnRNPC, hnRNPM, and SRSF1—are key regulators of RBFOX2-dependent splicing [41]. Together, these data implicate TEAD1 as a YAP-independent regulator of transcriptional and post-transcriptional programs in NEPC, potentially coordinating RNA splicing and DNA repair to sustain lineage plasticity.

## Discussion

With the advent of second-line therapies capable of inhibiting AR signaling, there has been a noted increase in the prevalence of treatment induced NEPC [1,42]. To date, there are limited data that detail the constituents and regulatory functions of the Hippo pathway in YAP-negative tumors like NEPC. However, a recent study by Han et al., using organoid models of NEPC trans-differentiation have shown that the YAP/TEAD pathway plays a key role in maintenance of ARPC lineage state, preventing tumor cells from undergoing the ARPC to NEPC lineage switch [43].

TEAD1 is considered a master regulator of subordinate transcription factors that regulate the expansion of pancreatic progenitors [44]. TEAD1 inhibits POL II binding and suppresses YAP1, LATS2, and TEAD3, and proliferation [45]. When TEAD1 is silenced, YAP1, LATS2, and TEAD3 all increase with a concomitant increase in proliferation. These studies highlight the executive function of TEAD1 in endocrine cells. In our study, in all three RNAseq datasets PANCREAS_BETA_CELLS is the highest hallmark pathway after E2F_TARGETS and G2M_CHECKPOINT separating ARPC and NEPC. Thus, we believe similar to pancreatic beta cells, that TEAD1 could be a master regulator of NEPC identity [44].

Strikingly, analyses of transcriptomic datasets of CRPC determined that YAP1 is not the only gene in the YAP/TAZ signaling pathway that is significantly altered in NEPC. There is a decrease in YAP1, TAZ, LATS2, and TEAD2 and an increase in LATS1, TEAD1 and RBFOX2 expression. In YAP positive cells, LATS1 and LATS2 suppress YAP activity. While closely related, these kinases have distinct transcriptional regulators and share common and unique substrates [46]. Therefore, unsurprisingly, while LATS2 is decreased, LATS1 expression is maintained/ increased in the NEPC tumors. Both LATS are associated with cell cycle regulation. LATS2 can promote p53 dependent checkpoints that can lead to cell cycle arrest or apoptosis [[46], [47], [48]]. Similar to the loss of p53 function in NEPC, the loss of LATS2 expression in NEPC may facilitate proliferation of the NEPC tumor phenotype [49,50]. Additionally, LATS2 can promote apoptosis through downregulating anti-apoptotic proteins, BCL2 and BCL-x(L), in human lung cancer cells [51]. Decreased LATS2 could allow for the high levels of expression of BCL2 observed in NEPC [52]. LATS1 expression is maintained or increased in NEPC tumors. LATS1 is a negative regulator of CDC2 and acts as a tumor suppressor regulating G2/M transition and apoptosis. Based on the data reported here, LATS1 may have a similar tumor suppressor role in NEPC [53,54]. TAZ was also decreased in NEPC. It is associated with YAP as a Hippo pathway effector and participates in the repression of proneuronal genes functioning as a repressor of neuronal differentiation [55].

TEAD family transcription factors are essential in mediating YAP-dependent gene expression [56]. TEAD1 activity is associated with tumor cell differentiation, proliferation and migration [45,[57], [58], [59], [60]]. Our data suggest that TEAD1 is highly expressed in the NEPC phenotype, with limited expression of TEAD2-4. The hypermethylation of YAP1 and hypomethylation of TEAD1 in PDX models and patient samples dictate epigenetic control of YAP1 and TEAD1 in treatment induced conversion from ARPC to NEPC, rather than transcriptional regulation. These events highlight the importance of TEAD1 in NEPC. We also observed that the YAP pathway is altered in NEPC impacting tumor cell differentiation, however, known YAP-TEAD inhibitors did not have a striking effect on NEPC cell number *in vitro*.

In addition to the significant alterations observed in the expression of YAP-pathway associated proteins in NEPC, we observed an increase in RBFOX2 transcript. RBFOX2 can splice in exon 6 in TEAD1. We determined that TEAD1 is spliced in both tumor models and patient samples of NEPC. Choi et al., have shown that RBFOX2 overexpression in the presence of YAP overexpression caused a dose-dependent increase in TEAD1-mediated transcriptional activity [12]. This activity was in the presence of YAP, the impact of this splicing event in unknown in NEPC in the absence of YAP.

Independent of TEAD1 splicing, the knockdown of RBFOX2 decreased the expression of SLC7A11 and the PI3K pathway in MSKCC EF1 cells. SLC7A11 promotes PI3K signaling in pituitary neuroendocrine tumors [61], gastric cancer [36], and pancreatic cancer [62] and RBFOX2 has already been shown to promote tumor growth by PI3K signaling in gastric cancer [37], suggesting RBFOX2 is central to PI3K signaling in NEPC. SLC7A11 also protects against ferroptosis [63,64]. This suggests that RBFOX2 may promote SLCA11 expression and ferroptosis resistance in NEPC [65].

We expected that TEAD1/TEAD1Δ6 must interact with a cofactor to drive transcription in NEPC. Using RIME to capture transcriptional co-factors and chromatin-associated proteins we identified VGLL4 as a possible interacting factor from the original 428 proteins identified, but predominantly identified splicing and DNA damage repair associated proteins. VGLL4 negatively regulates the TEAD1-YAP1 transcriptional complex however it is unknown what its function is in YAP-negative NEPC [66]. Another transcription factor of interest identified through RIME was EBF3 a transcription factor associated with glioma and glioblastoma [67,68]. Strikingly, the TEAD1 interactome suggested interactions with the spliceosome and DNA damage repair enzymes associated with the activities of the splicing factor RBFOX2 [41] in MSKCC EF1 cells. These findings and the identification of the incorporation of exon 6 into the TEAD1 gene highlight the overlap and interactions between the spliceosome and TEAD1 in MSKCC EF1 cells.

To determine the impact of TEAD1/ TEAD1Δ6 on gene expression and differentiation in NEPC, we knocked down TEAD1 in MSKCC EF1 cells. The re-expression of epithelial-associated genes in the MSKCC EF1 TEAD1 knockdown cells suggest TEAD1 can suppress the epithelial phenotype in NEPC.

INSM1 is a transcriptional repressor and well characterized protein expressed in NEPC. An inverse correlation of INSM1 and YAP expression distinguishes ARPC from NEPC [6,69]. INSM1 is expressed at high levels in MSKCC EF1 cells. INSM1 can cooperate with TEAD1 to repress the transcription of TEAD1-targeted genes [70]. Our data point to TEAD1 as another transcriptional master regulator that may interact with INSM1 in NEPC to maintain the neuroendocrine phenotype by suppressing the epithelial phenotype. These findings warrant further investigation.

In conclusion, this study highlights how the epigenetic silencing of YAP and the YAP-pathway can modify TEAD1 interactions and activity and control downstream gene expression, promoting a treatment induced neuroendocrine phenotype in prostate cancer.

## CRediT authorship contribution statement

**Lisha G. Brown:** Writing – review & editing, Validation, Methodology, Investigation, Data curation. **Ilsa M. Coleman:** Writing – review & editing, Methodology, Investigation, Formal analysis, Data curation. **Tony L.H. Chu:** Writing – review & editing, Methodology, Investigation, Formal analysis, Data curation. **Erolcan Sayar:** Writing – review & editing, Methodology, Investigation, Formal analysis. **Radhika A. Patel:** Writing – review & editing, Methodology, Investigation. **Brian Hanratty:** Writing – review & editing, Methodology, Investigation, Formal analysis, Data curation. **Mohamed Adil:** Writing – review & editing, Visualization, Methodology, Investigation, Formal analysis, Data curation. **Dapei Li:** Writing – review & editing, Methodology, Investigation. **Yongtao Li:** Writing – review & editing, Methodology, Investigation. **Holly M. Nguyen:** Writing – review & editing, Supervision, Resources, Methodology, Investigation. **Conner J. Sessions:** Writing – review & editing, Methodology, Investigation. **Erin L. Sweeney:** Writing – review & editing, Methodology, Investigation. **Joshi J. Alumkal:** Writing – review & editing, Supervision, Methodology, Investigation, Funding acquisition. **Rui M. Gil da Costa:** Writing – review & editing, Methodology, Investigation, Formal analysis, Data curation. **Yuzhuo Wang:** Writing – review & editing, Resources. **Daniel W. Lin:** Writing – review & editing, Resources, Funding acquisition. **Lawrence D. True:** Writing – review & editing, Resources, Methodology, Investigation, Formal analysis, Data curation. **Ruth Dumpit:** Writing – review & editing, Methodology, Investigation, Formal analysis, Data curation. **Eva Corey:** Writing – review & editing, Resources, Investigation, Funding acquisition. **John K. Lee:** Writing – review & editing, Resources, Project administration, Methodology, Investigation. **Peter S. Nelson:** Writing – review & editing, Resources, Project administration, Methodology, Investigation, Funding acquisition. **Li Xin:** Writing – review & editing, Supervision, Methodology, Investigation, Formal analysis. **Michael C. Haffner:** Writing – review & editing, Supervision, Resources, Project administration, Methodology, Investigation, Funding acquisition, Formal analysis, Conceptualization. **Colm Morrissey:** Writing – review & editing, Writing – original draft, Supervision, Resources, Project administration, Investigation, Funding acquisition, Conceptualization.

## Declaration of competing interest

EC has obtained funding from Genentech, Sanofi, AbbVie, Astra Zeneca, Foghorn Pharmaceuticals, Kronos Bio, MacroGenics, Janssen Research, Bayer Pharmaceuticals, Forma Pharmaceuticals, Gilead, Zenith Epigenetics, BoundlesBio, and was a consultant for DotQuant, all unrelated to the present work. PSN has received fees for advisory work from BMS, Genentech, AstraZeneca, and research support from Janssen for studies unrelated to the present work. CM has obtained funding from Janssen Research, Astra Zeneca, Novartis, and Genentech unrelated to the present work. LDT is a Cofounder and holder of equity in Alpenglow Biosciences unrelated to the present work. JKL has received research funding from Immunomedics and serves as a scientific advisor for and has equity in PromiCell Therapeutics. MCH served as a paid consultant/received honoraria from Pfizer and AstraZeneca and has received research funding from Merck, Novartis, Genentech, Promicell and Bristol Myers Squibb. M Adil is consultant for Quest Diagnostics, Inc. JJA has received consulting fees from Fortis Therapeutics and ORIC Pharmaceuticals and research support to his institution from Beactica, Zenith Epigenetics, Astellas Pharma Global Development, Inc., and Pfizer, Inc. outside of the present work.

## Appendix Supplementary materials

Image, application 1

Image, application 2

Image, application 3

Image, application 4

Image, application 5

Image, application 6
