# Supplementary material for: Systematic analysis of hippo pathway signaling identifies TEAD1 as a transcriptional regulator of neuroendocrine prostate cancer
Source: Neoplasia. 2026 May 30;78:101321. doi: 10.1016/j.neo.2026.101321 (PMC13241777; doi:10.1016/j.neo.2026.101321)

**Supplemental Figure 1. Gene expression and pathway analysis of ARPC and NEPC CRPC RNAseq datasets.** (A) The number of AR+/NE-(ARPC) and AR-/NE+ (NEPC) specimens in each of the datasets. (B through D) Volcano plots comparing AR+/NE-(ARPC) and AR-/NE+ (NEPC) specimens in LuCaP models, SU2C, and UW TAN specimens. (E) GSEA of Hallmark Pathways altered in NEPC compared to ARPC in LuCaP, SU2C, and UW TAN specimens. GSEA summary plots filtered for at least one comparison with FDR<0.05 (sorted by mean NES – Normalized enrichment score).


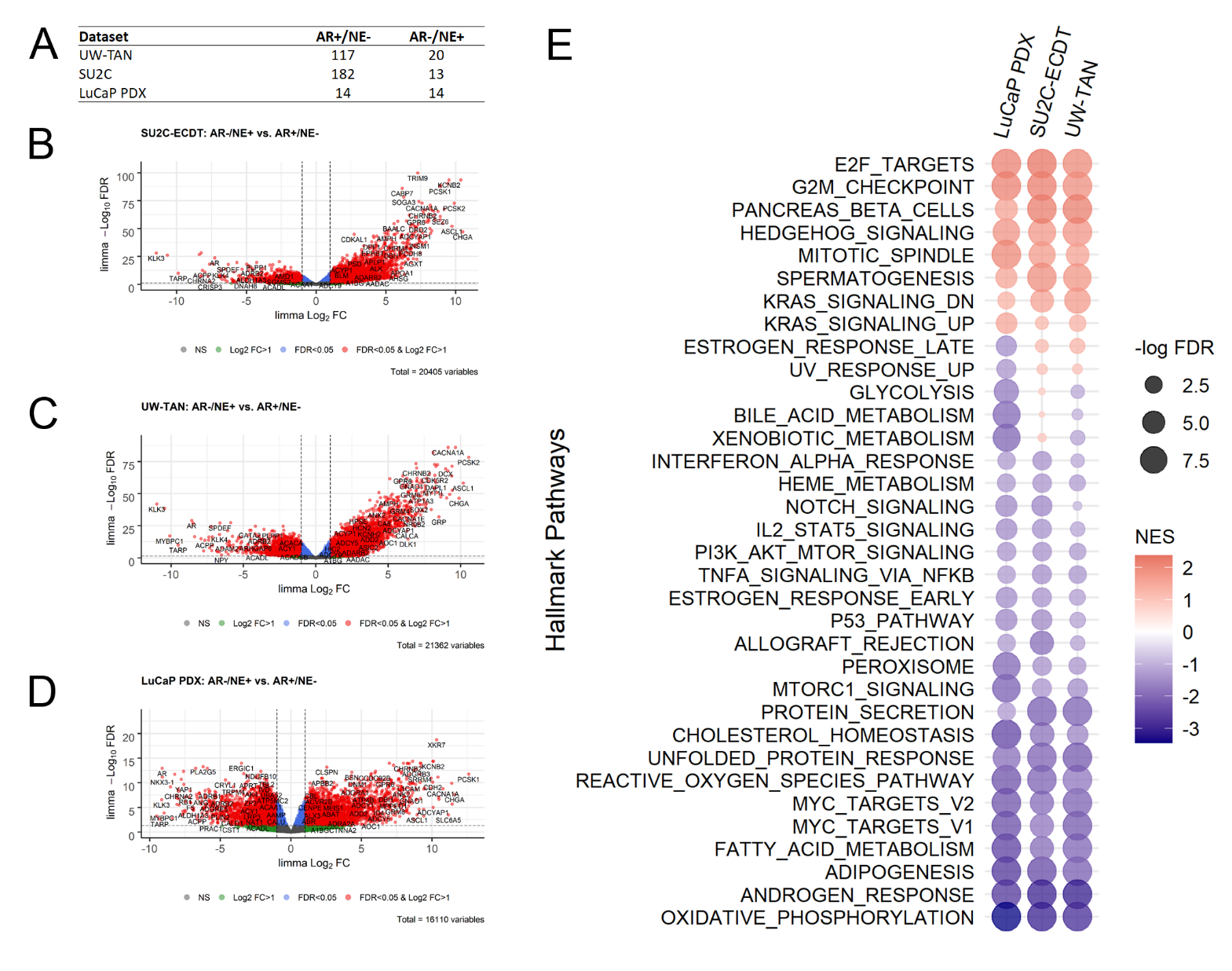


**Supplemental Figure 2. Biomarkers of NEPC in PDX models and patient specimens.** Transcript abundance in (A) 45 LuCaP PDX models, (B) 270 biopsies from the SU2C dataset, and (C) 132 metastases from 77 patients from the UW TAN program. Androgen receptor positive (ARPC; green) and (NEPC; orange). **** p<0.0001.


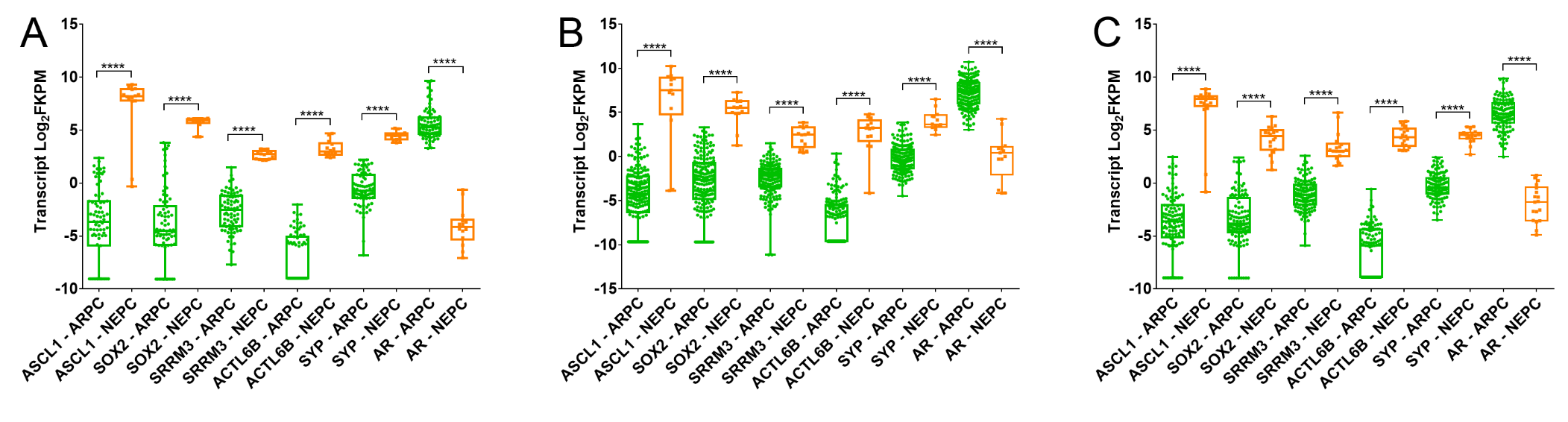


**Supplemental Figure 3. Verifying the NEPC phenotype in LuCaP xenografts.** *In vivo* examples of NEPC (LuCaP 208.2, 208.1, 173.1, 145.2, 145.1, 93, and 49) and ARPC (LuCaP 170.1, 147, 136, 96, 70, 35, and 23.1) PDX models. Highlighting the expression of the ARPC marker (A) AR, and NEPC markers (B) ASCL1, (C) SYP, (D) BAF53B, and (E) SOX2 by IHC. Bar = 20 microns.


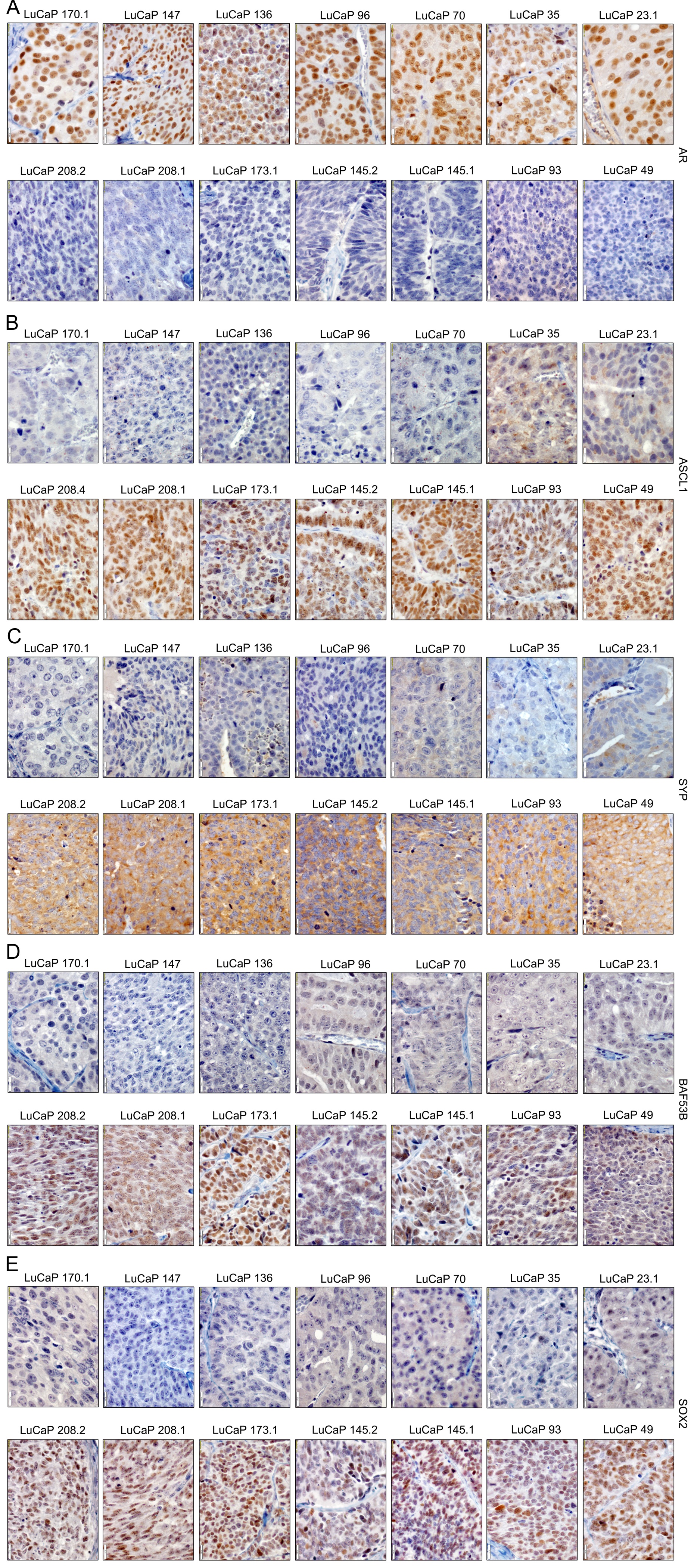


**Supplemental Figure 4.** (A) Protein expression of YAP and TAZ in prostate cancer ARPC and NEPC cell lines. (B) A luciferase assay of YAP/TAZ activity in MSKCC EF1 (NEPC) cells and control 293T epithelial cells.


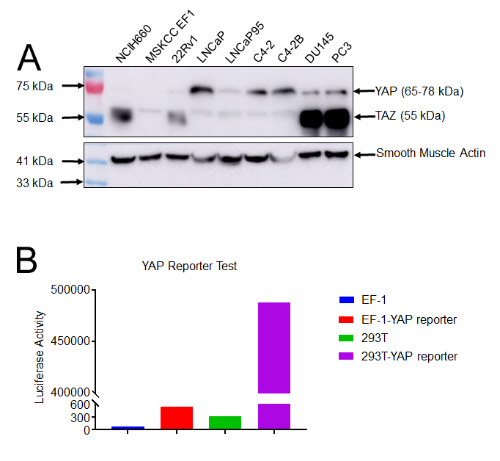


**Supplemental Figure 5. YAP-associated gene expression in cell lines and models.** RNAseq heat map of phenotype- and YAP-associated gene expression in ARPC and NEPC. (A) LuCaP PDX models, (B) cell lines, and (C) the LTL331 line before and after castration *in vivo*. Results are expressed as log_2_ Fragments Per Kilobase of transcript per Million mapped reads (FPKM) and colored according to scale.

**Supplemental Figure 6. snRNAseq of ARPC and NEPC patient liver metastases.** tSNE plot of two phenotypic markers and YAP-pathway associated genes from two ARPC and two NEPC liver metastasis from each of four prostate cancer patients. Gene expression values are log-normalized; values above the 95^th^ percentile were capped for visualization on the UMAP.


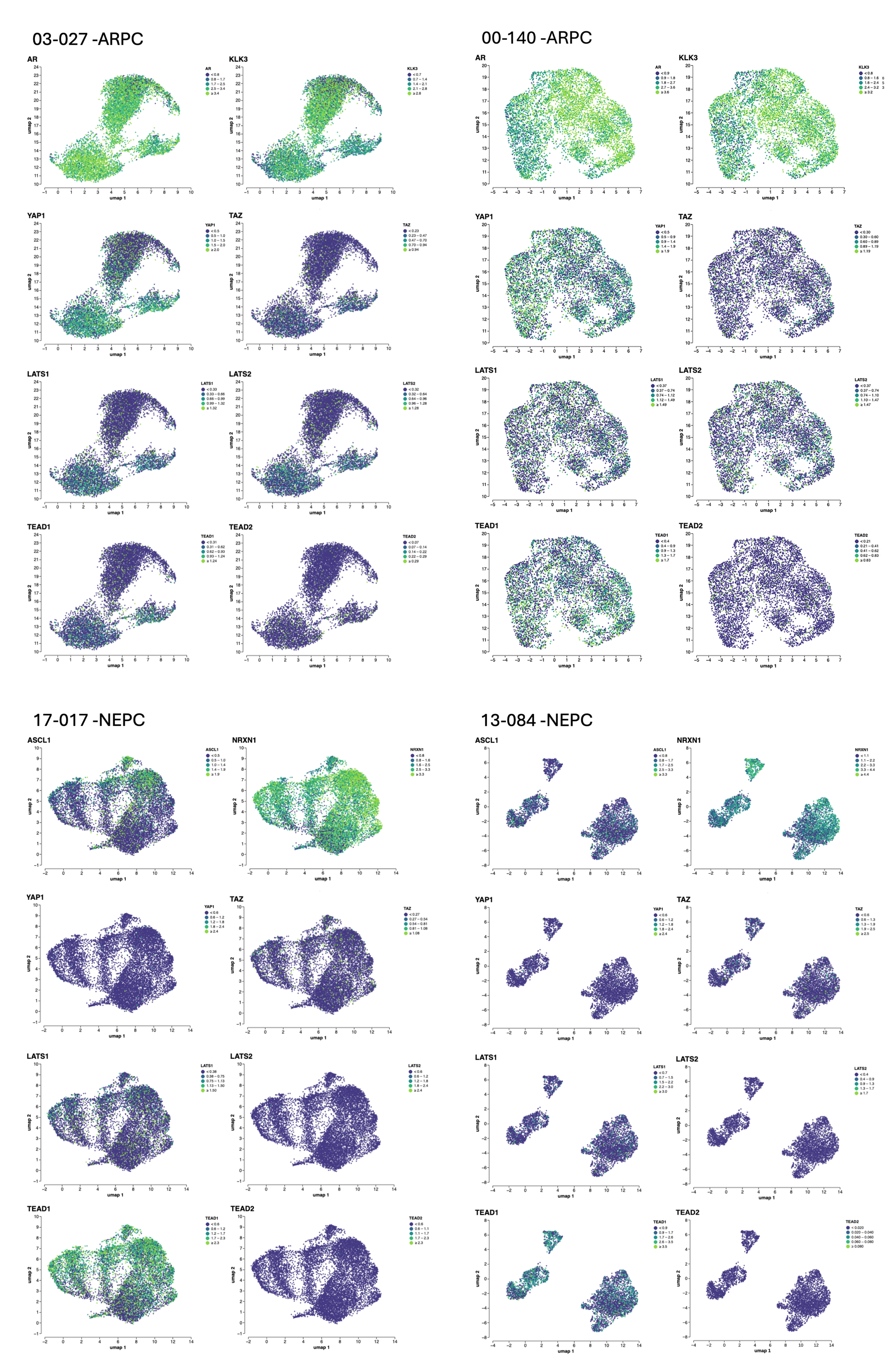


**Supplemental Figure 7. LATS1 suppresses proliferation in NEPC.** NCI-H660, LuCaP 145.2, and MSKCC EF1 (NEPC) cells were treated with 1-10 μM LATS-IN-1 for 72 h and assessed for viability (3 replicates). * p<0.05; ** p<0.01; *** p<0.001; **** p<0.0001.


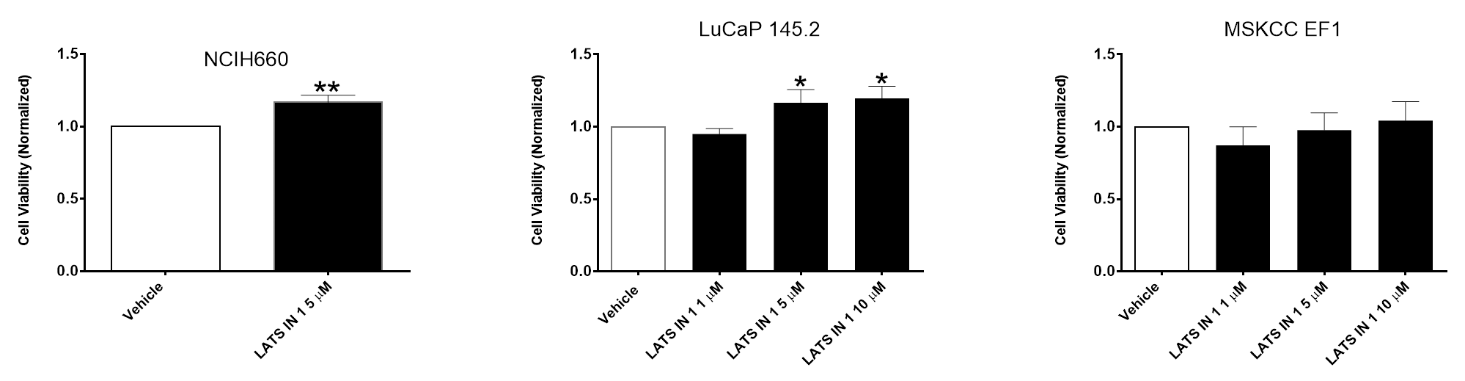


**Supplemental Figure 8. TEAD1 is the primary TEAD regulating gene expression in NEPC.** TEAD1-4 transcript levels in (A) 45 LuCaP PDX models, (B) 270 biopsies from the SU2C dataset, and (C) 132 metastases from 77 patients from the UW TAN program. Androgen receptor positive (ARPC; green) and (NEPC; orange). * p<0.05; ** p<0.01; *** p<0.001; **** p<0.0001.


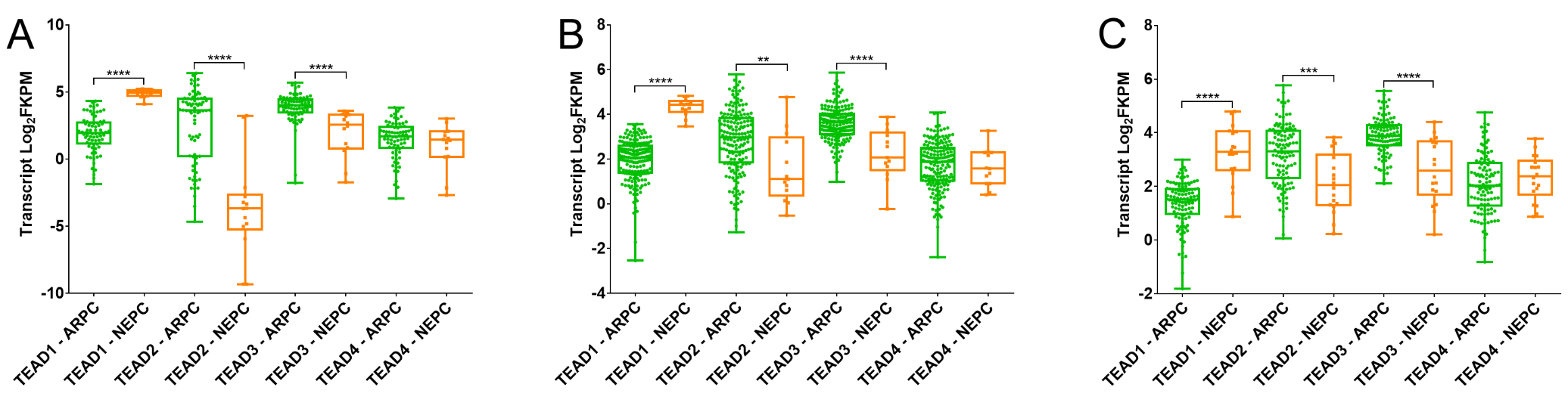


**Supplemental Figure 9. TEAD1 is present in the nucleus of NEPC models.** YAP and TEAD1 expression in (A) ARPC (LuCaP 23.1, 35, 70, 96, 136, 147, and 170.1) and (B) NEPC (LuCaP 49, 93, 145.1, 145.2, 173.1, 208.1, and 208.2) PDX models. (C) Western analysis of a siRNA of knockdown of *YAP1* and *TEAD1* in DU145 cells with actin control to verify antibody specificity. Bar = 20 microns.


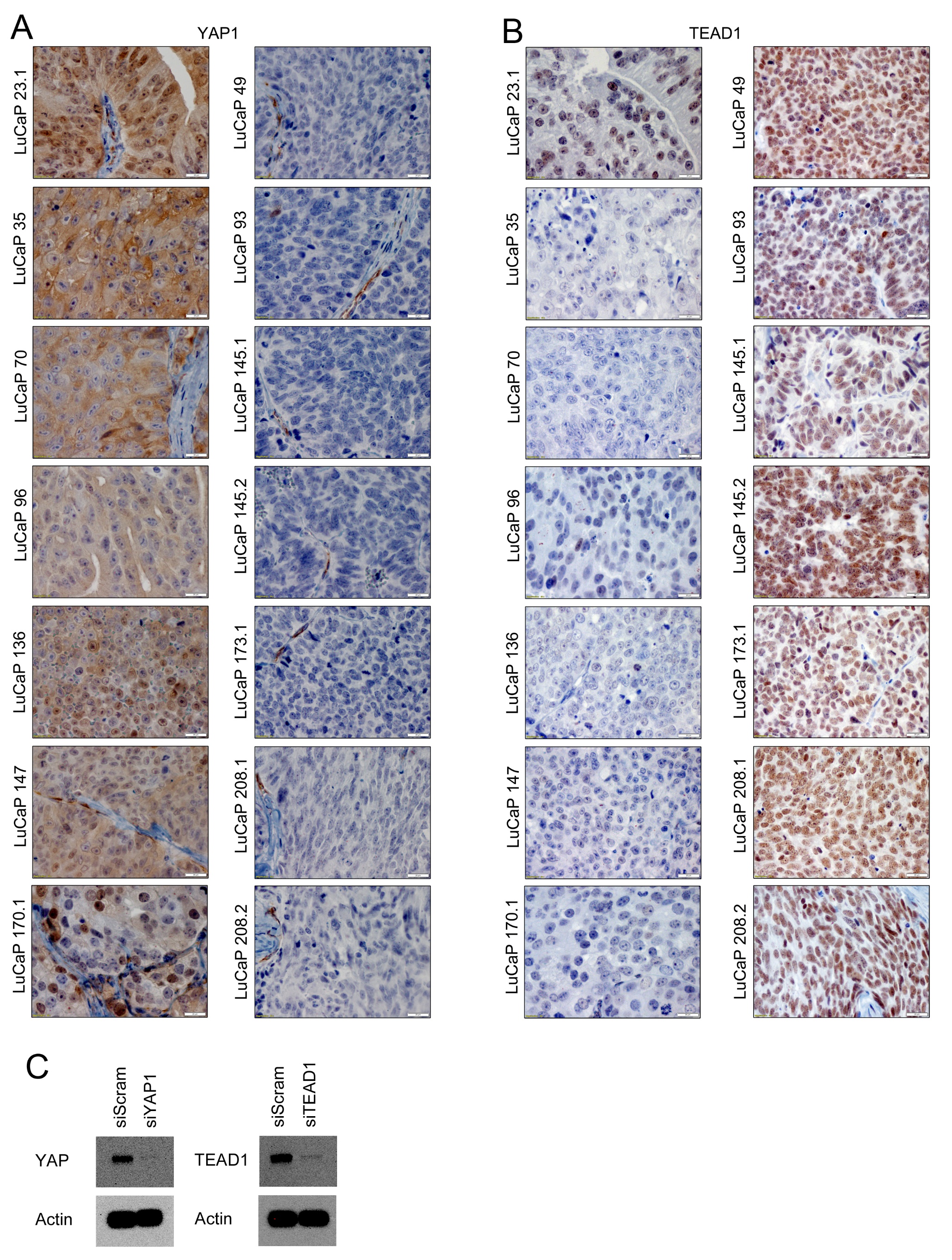


**Supplemental Figure 10. RBFOX2 transcript increases in NEPC.** RBFOX family transcript abundance in (A) 45 LuCaP PDX models, (B) 270 biopsies from the SU2C dataset, and (C) 132 metastases from 77 patients from the UW TAN program. Androgen receptor positive (ARPC; green) and (NEPC; orange). ** p<0.01; *** p<0.001; **** p<0.0001.


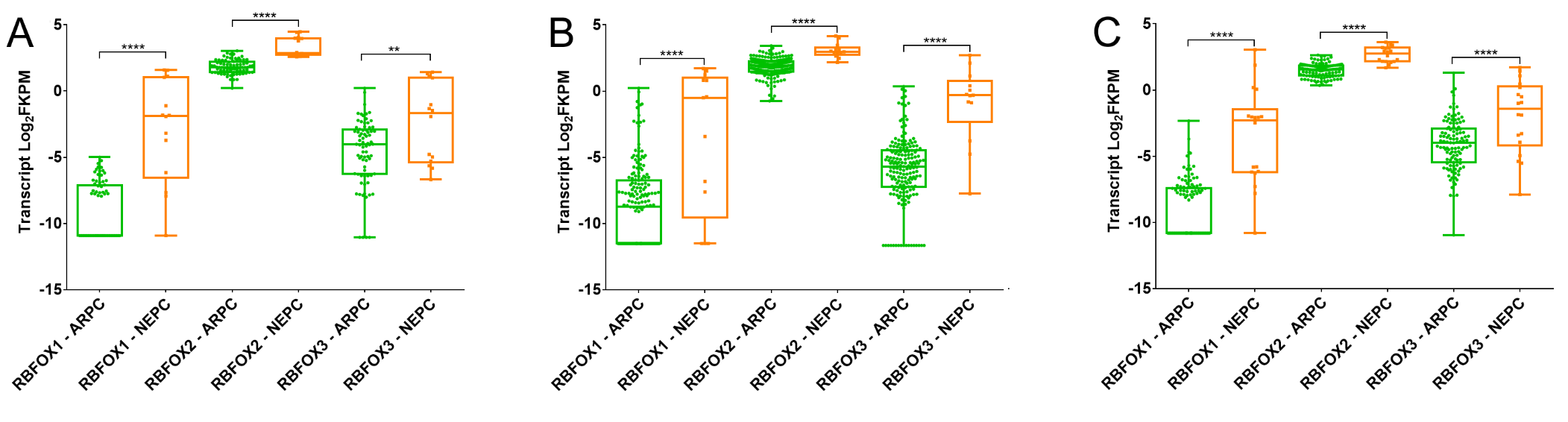


**Supplemental Figure 11. Silencing RBFOX2 decreases SLC7A11 and PI3K signaling in NEPC.** (A) Differential gene expression of protein coding genes following siRBFOX2 knockdown in MSKCC EF1 cells with a log_2_FPKM > 0 and a fold difference in expression >1. (B) GSEA of the KEGG pathways in RBFOX2 knockdown MSKCC EF1 cells.


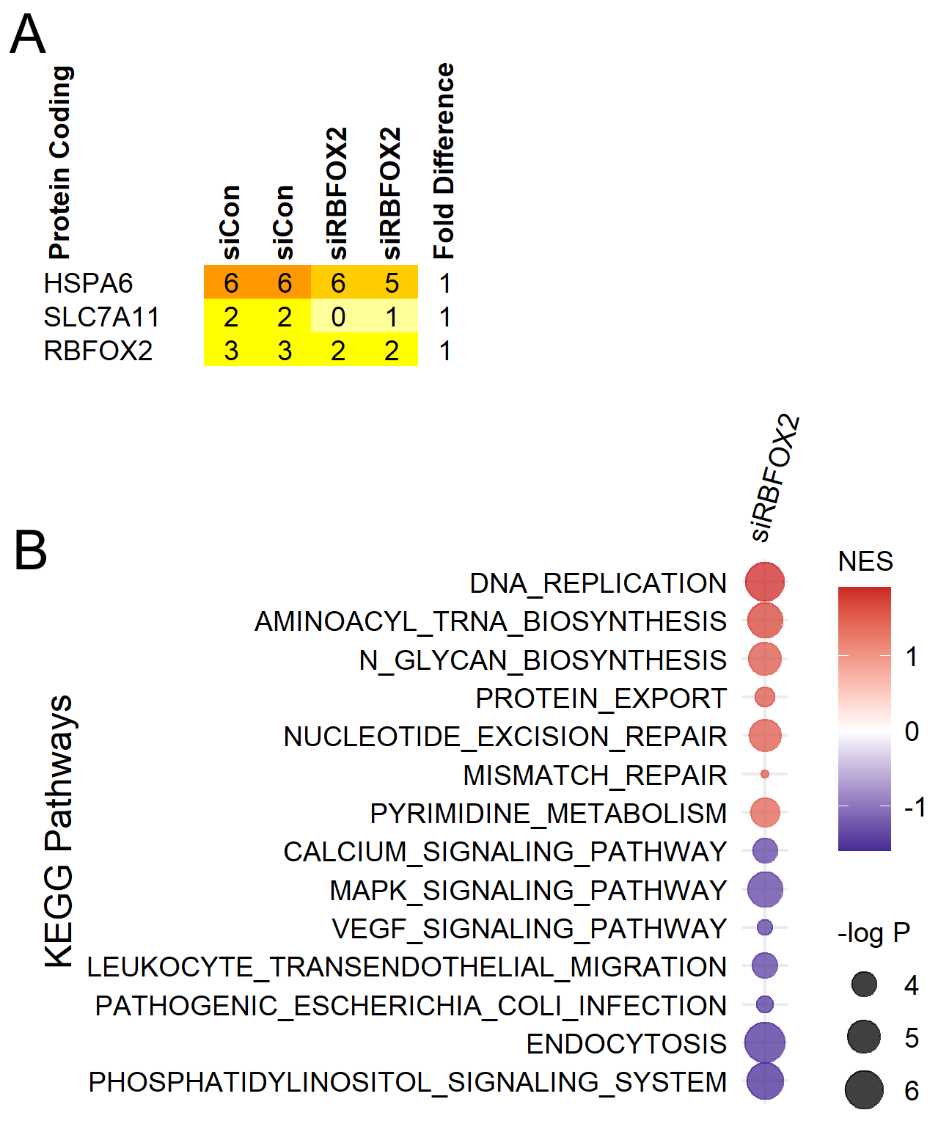


**Supplemental Figure 12. No effective TEAD1 inhibitors that block TEAD1 activity in the absence of YAP1.** Relative cell viability of NCI-H660, LuCaP 145.2, and MSKCC EF1 cells after 72 h treatment with TEAD1 inhibitors VT103, K975, and MYF-01-37 at a concentration of 1-10 μM *in vitro*. * p<0.05; ** p<0.01.


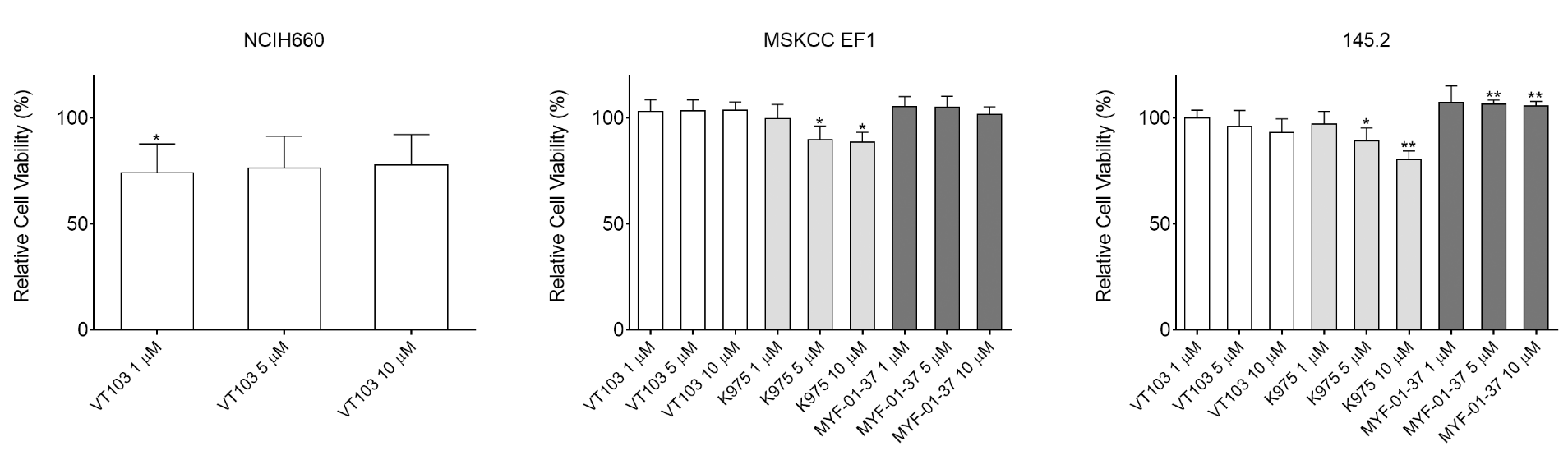


**Supplemental Figure 13. The spliceosome is interactive with TEAD1 in MSKCC EF1 cells.** (A) Overlap of siTEAD1 altered genes in MSKCC-EF1 (FC>3) and NCI-H660 (FC>3) cells. (B) Changes in Hallmark Pathways after TEAD1 knockdown with a P-value<0.05 in at least one dataset in MSKCC EF1 and NCI-H660 cells. (C) Expression of neuroendocrine-associated genes (log_2_FPKM), after TEAD1 knockdown in MSKCC EF1 and NCI-H660 cells.


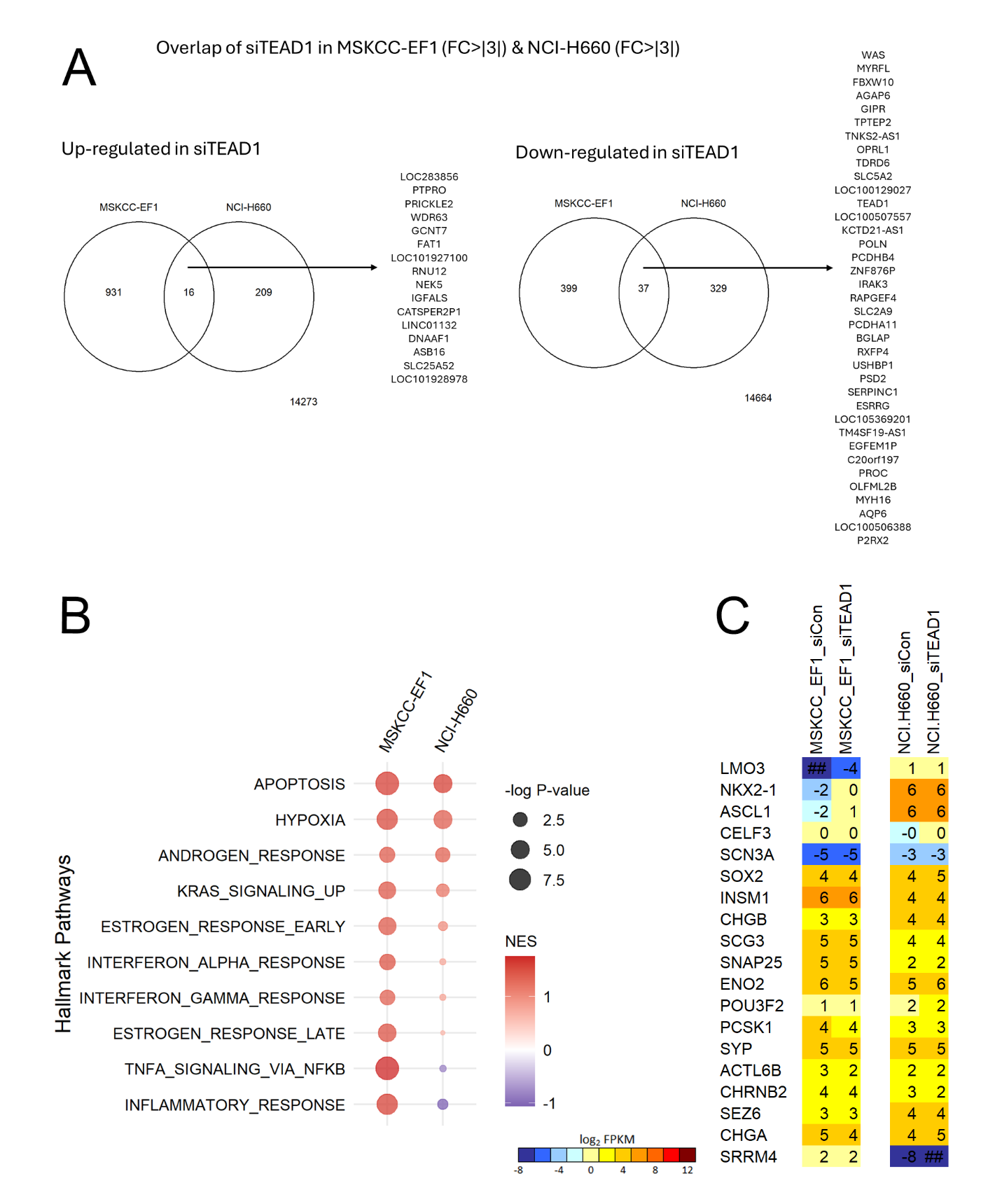


**Supplemental Figure 14. The spliceosome is interactive with TEAD1 in MSKCC EF1 cells.** (A) Immunoprecipitation of TEAD1 using an hnRNP U antibody in MSKCC EF1 cells. (B) Enrichr analysis of proteins identified as part of the TEAD1 interactome through RIME in MSKCC EF1 cells. Eighty-six genes were assessed by Enrichr and associated with RNA splicing in the GO Biological Process 2025.


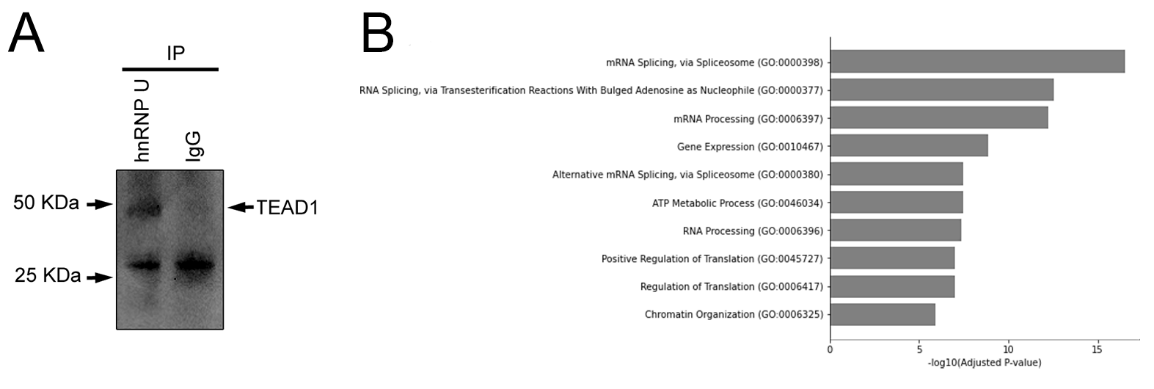

Supplement: Supplementary file 1 [file mmc1.docx]
