# Supplementary figures and images for: Systematic analysis of hippo pathway signaling identifies TEAD1 as a transcriptional regulator of neuroendocrine prostate cancer
Source: Neoplasia. 2026 May 30;78:101321. doi: 10.1016/j.neo.2026.101321 (PMC13241777; doi:10.1016/j.neo.2026.101321)

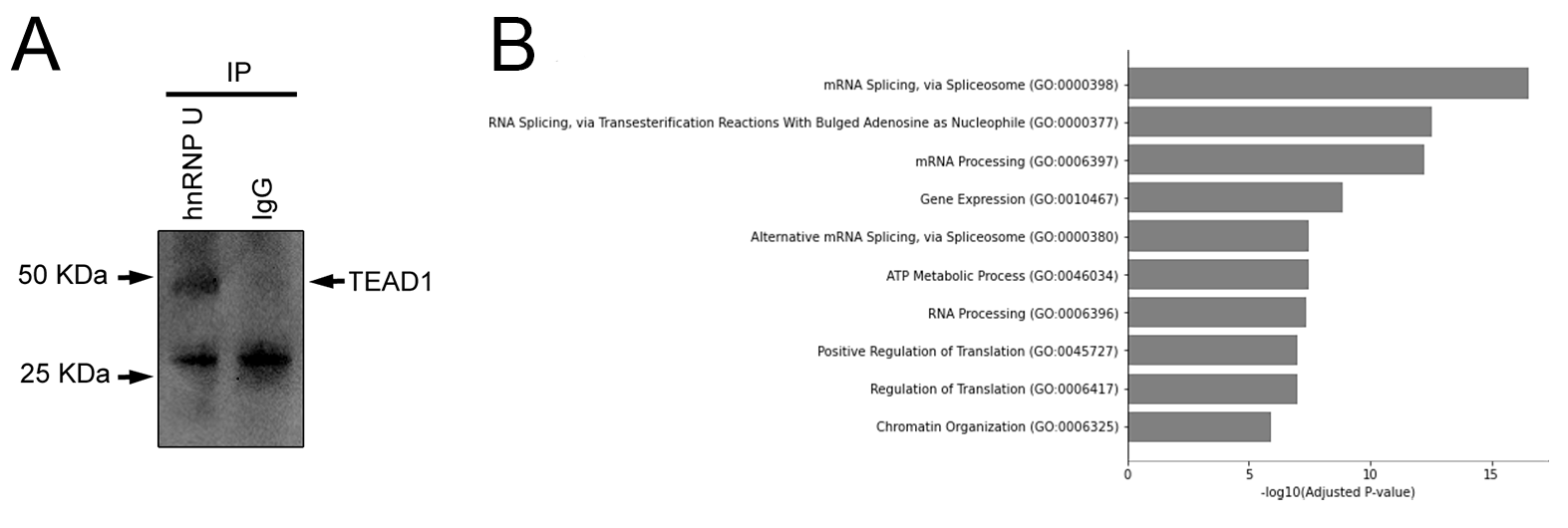

Supplement: Supplementary file 6 [file mmc6.zip › Supplemental Figure 14.tif]

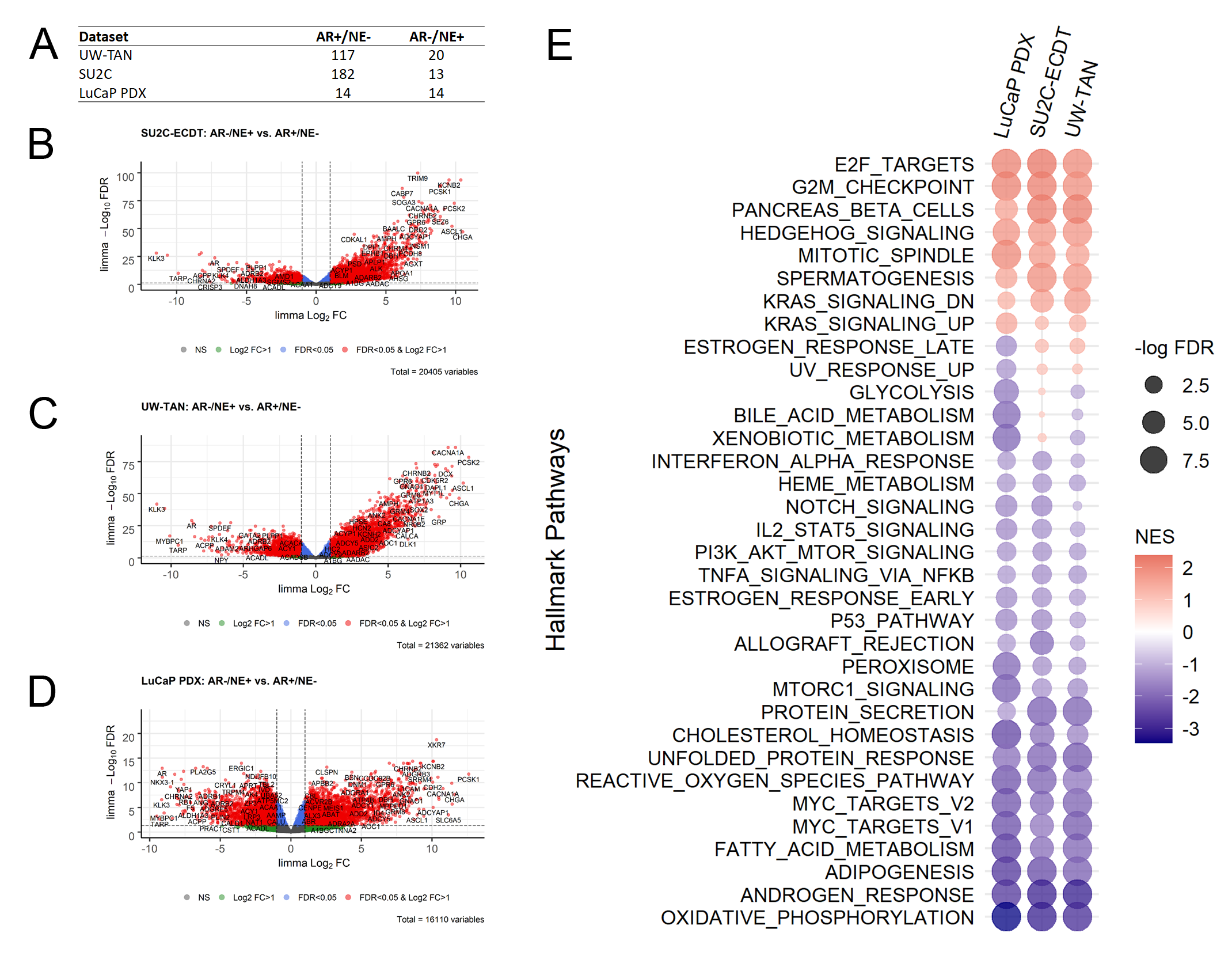

Supplement: Supplementary file 6 [file mmc6.zip › Supplemental Figure 1.tif]

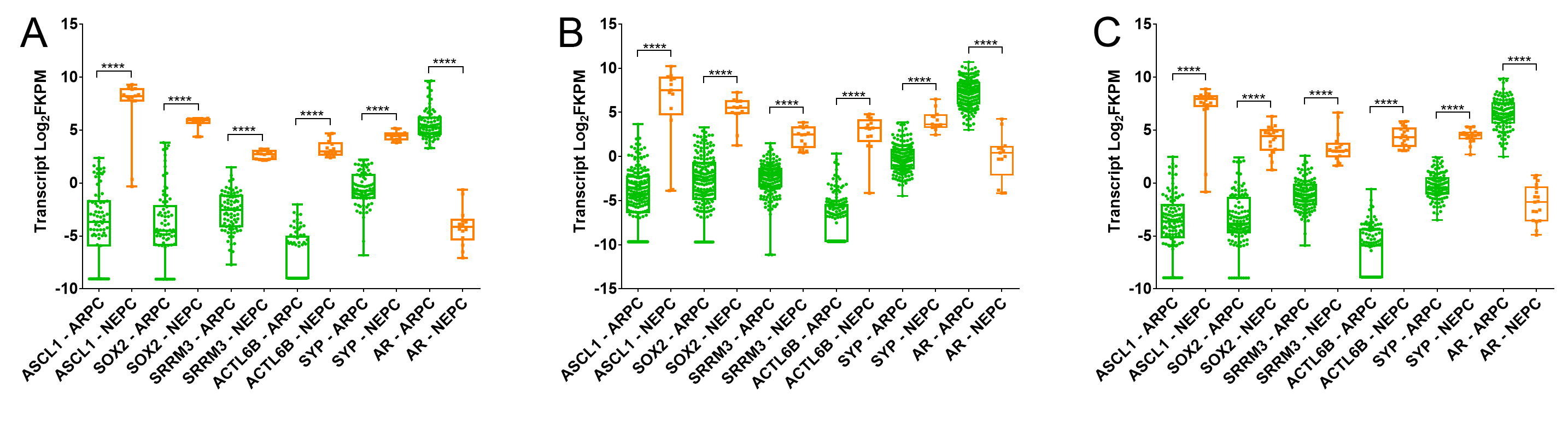

Supplement: Supplementary file 6 [file mmc6.zip › Supplemental Figure 2.tif]

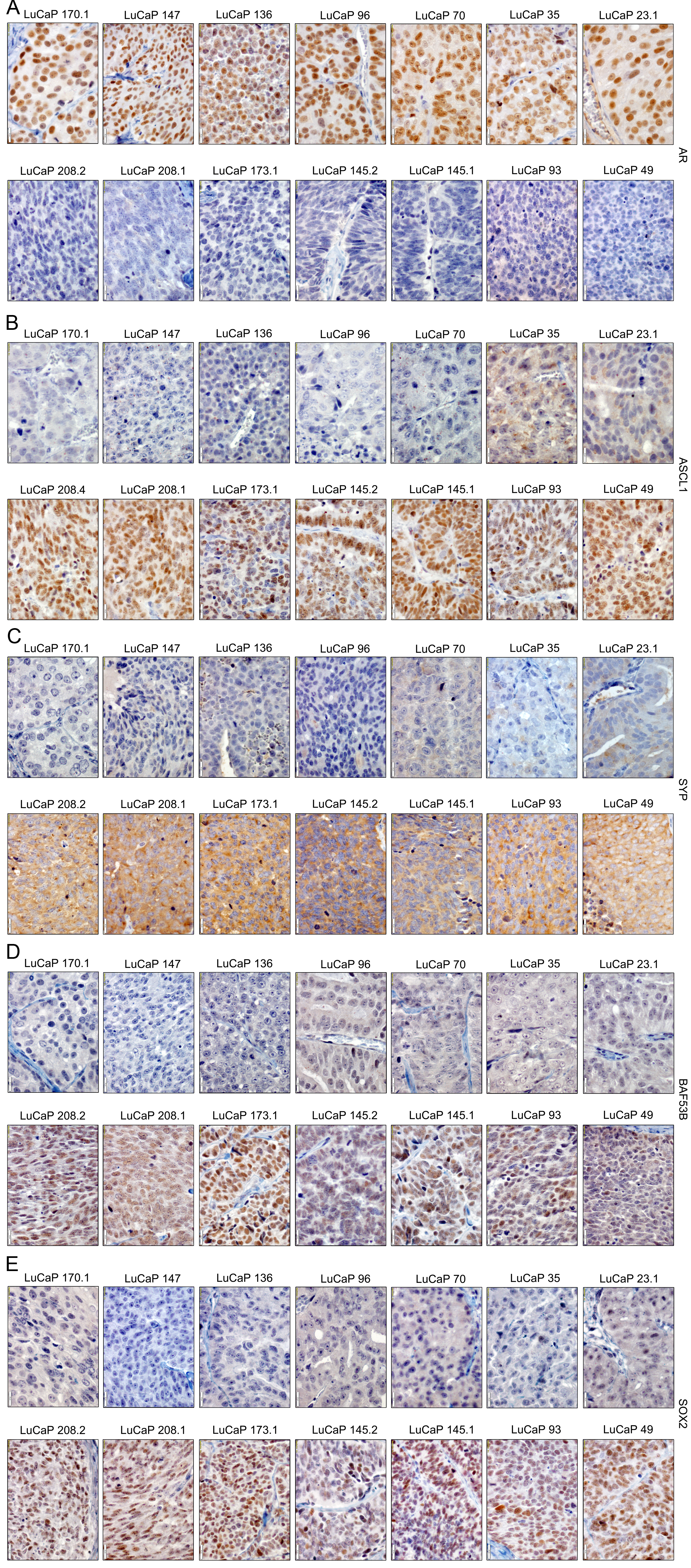

Supplement: Supplementary file 6 [file mmc6.zip › Supplemental Figure 3.tif]

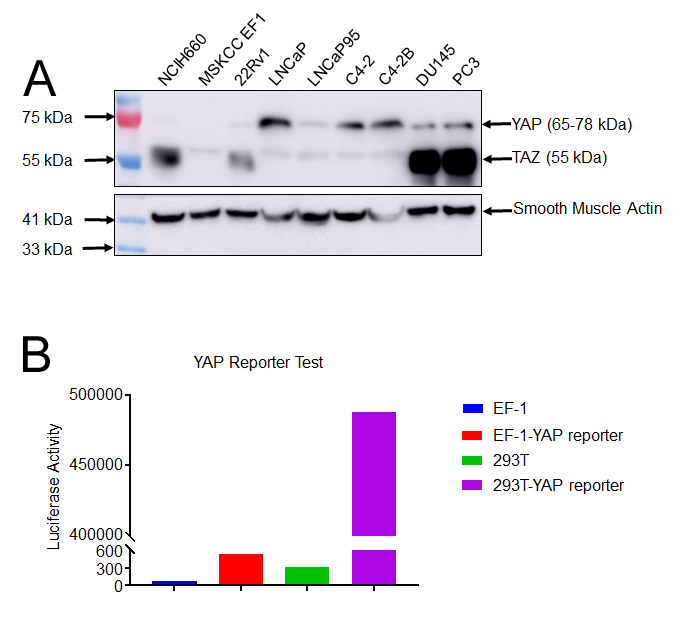

Supplement: Supplementary file 6 [file mmc6.zip › Supplemental Figure 4.tif]

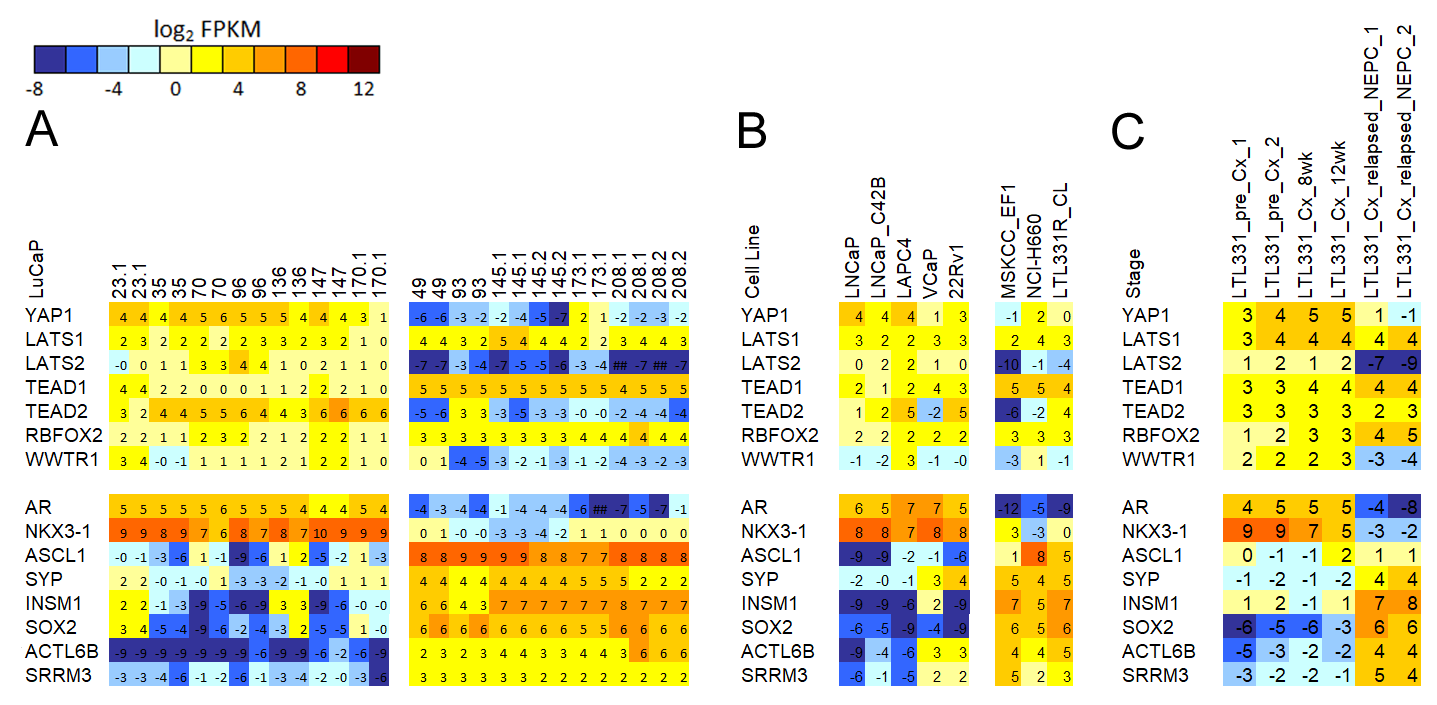

Supplement: Supplementary file 6 [file mmc6.zip › Supplemental Figure 5.tif]

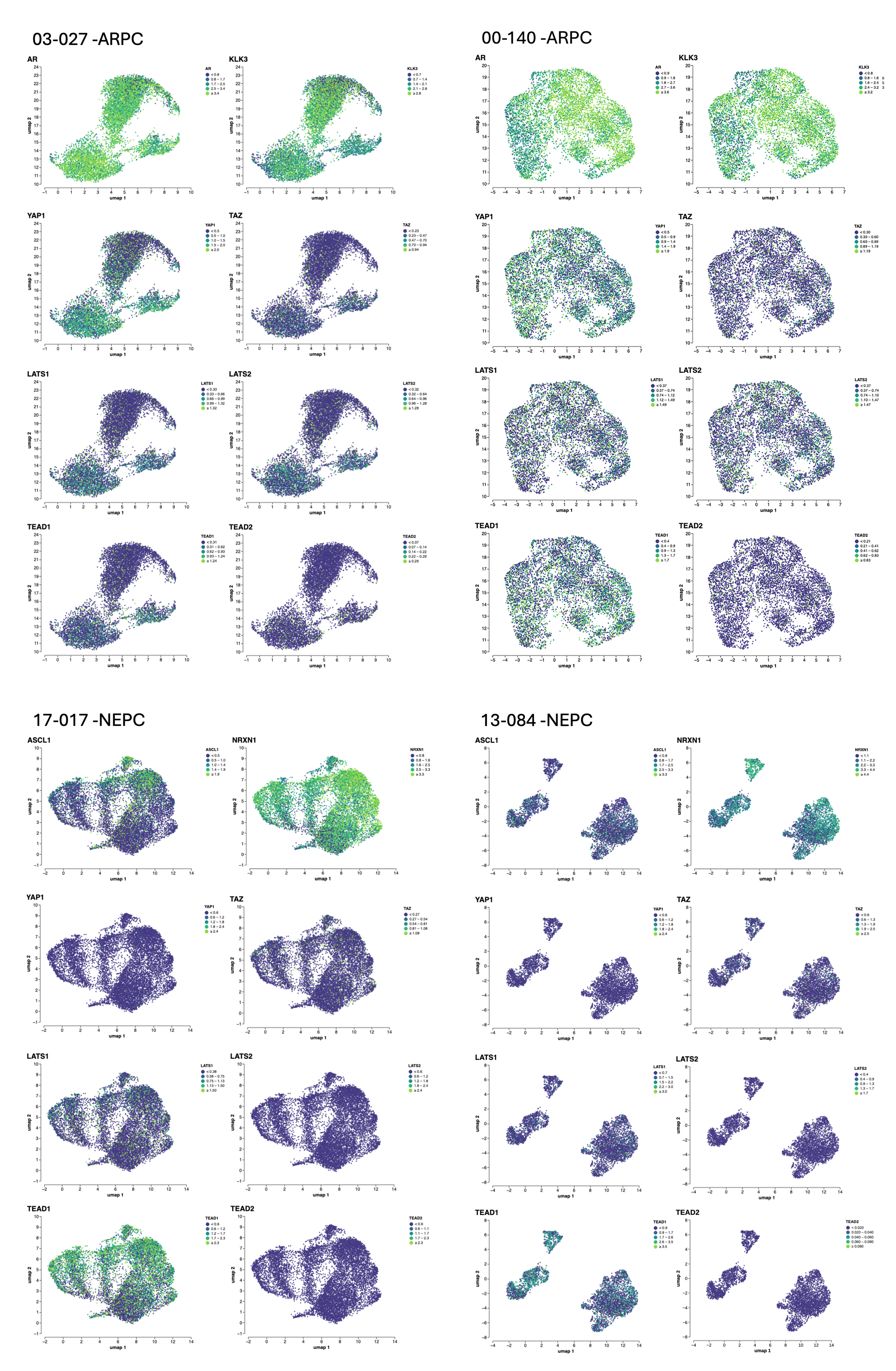

Supplement: Supplementary file 6 [file mmc6.zip › Supplemental Figure 6.tif]

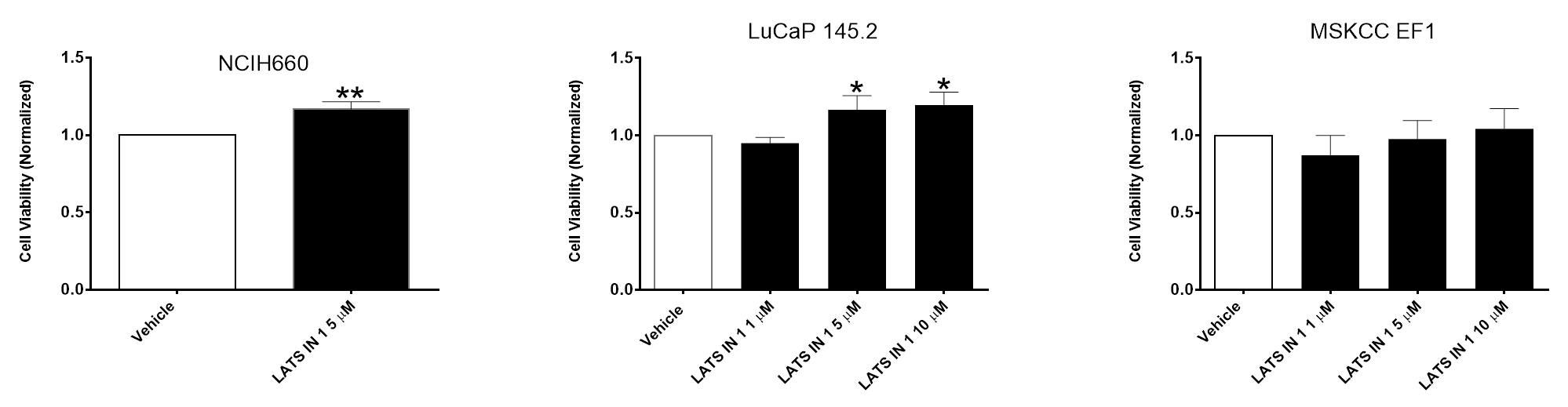

Supplement: Supplementary file 6 [file mmc6.zip › Supplemental Figure 7.tif]

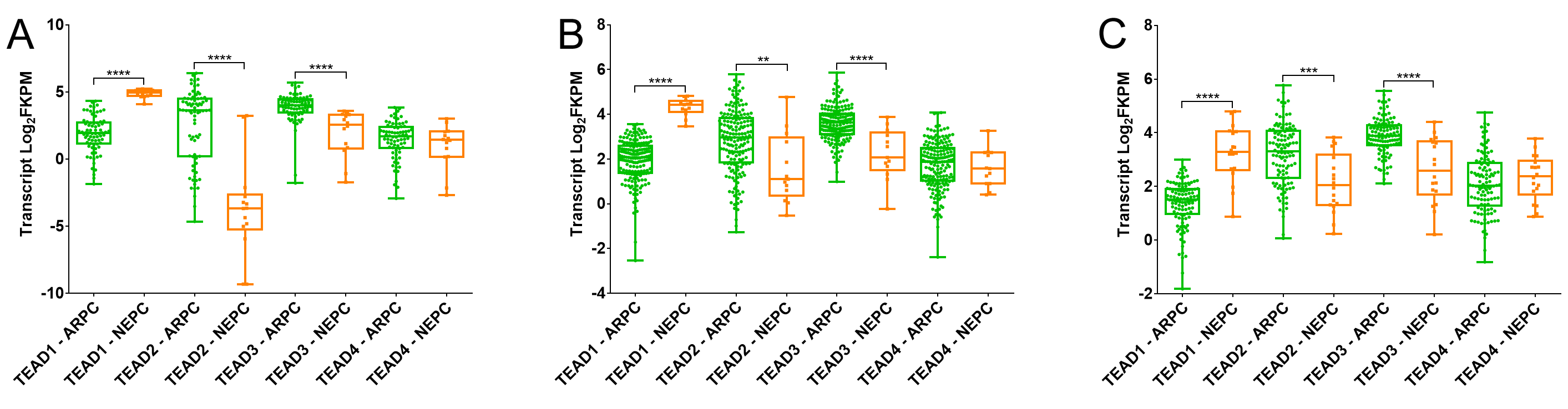

Supplement: Supplementary file 6 [file mmc6.zip › Supplemental Figure 8.tif]

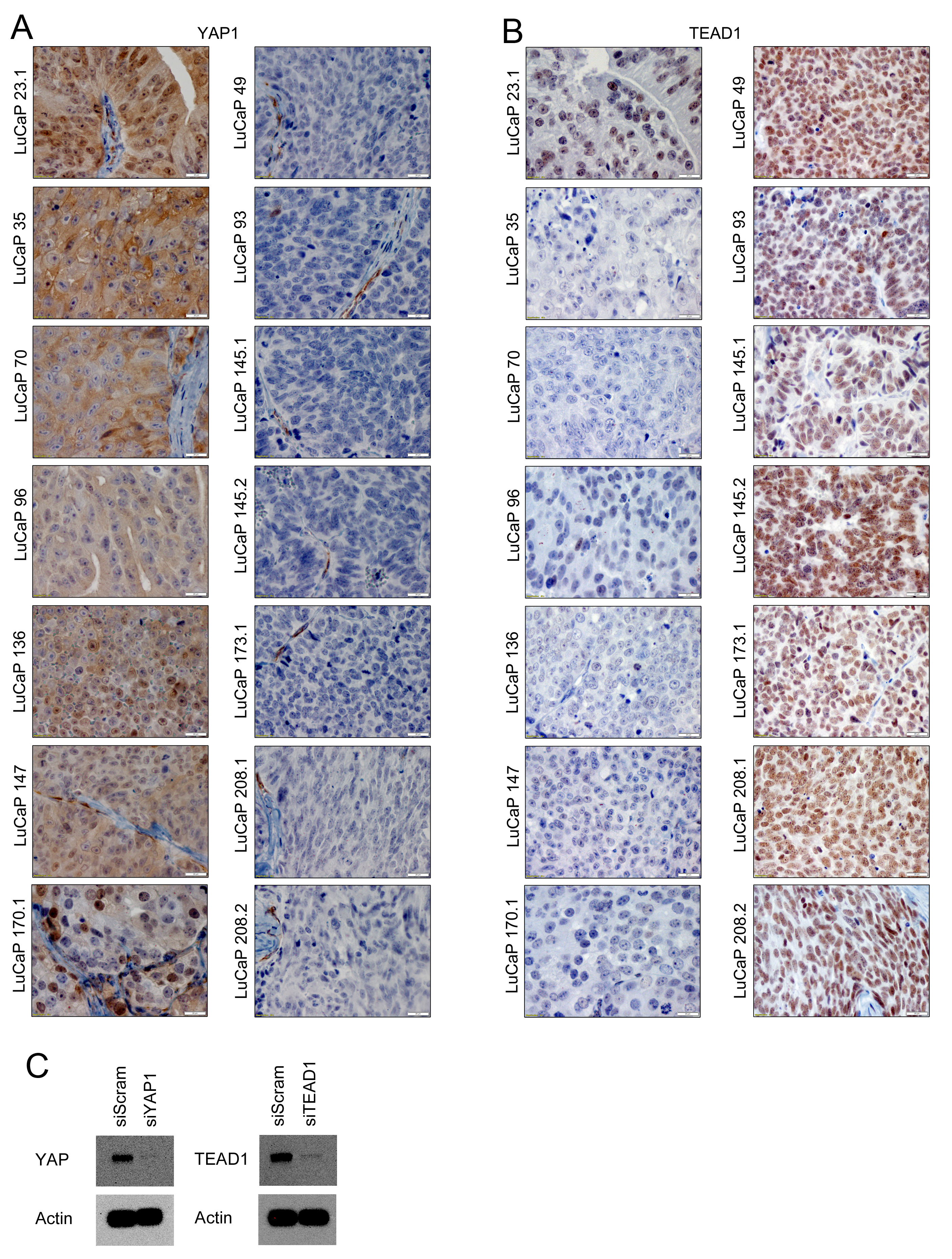

Supplement: Supplementary file 6 [file mmc6.zip › Supplemental Figure 9.tif]

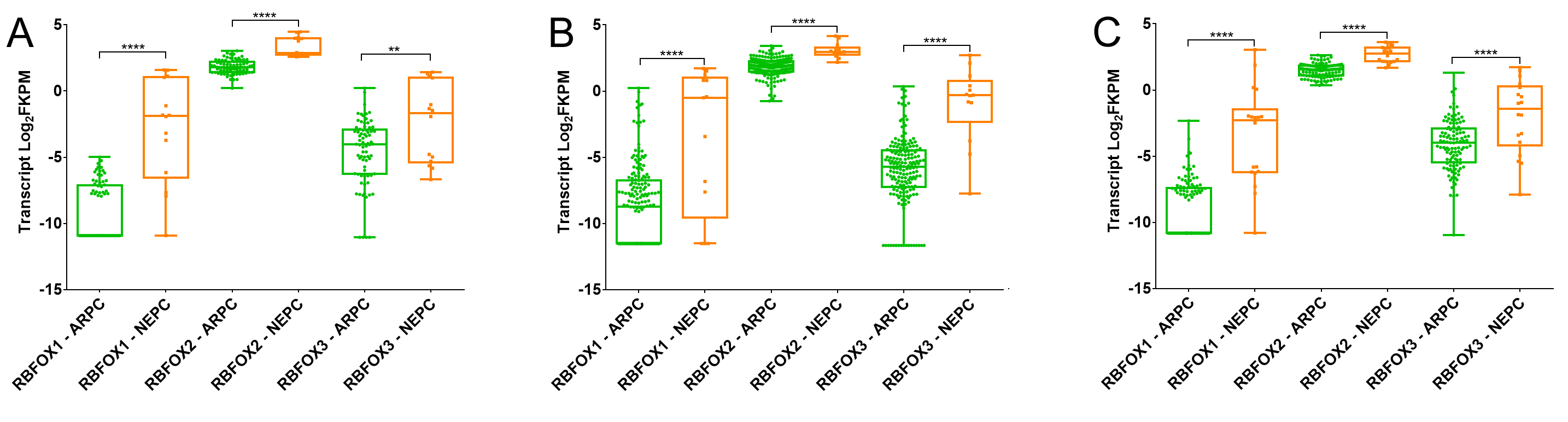

Supplement: Supplementary file 6 [file mmc6.zip › Supplemental Figure 10.tif]

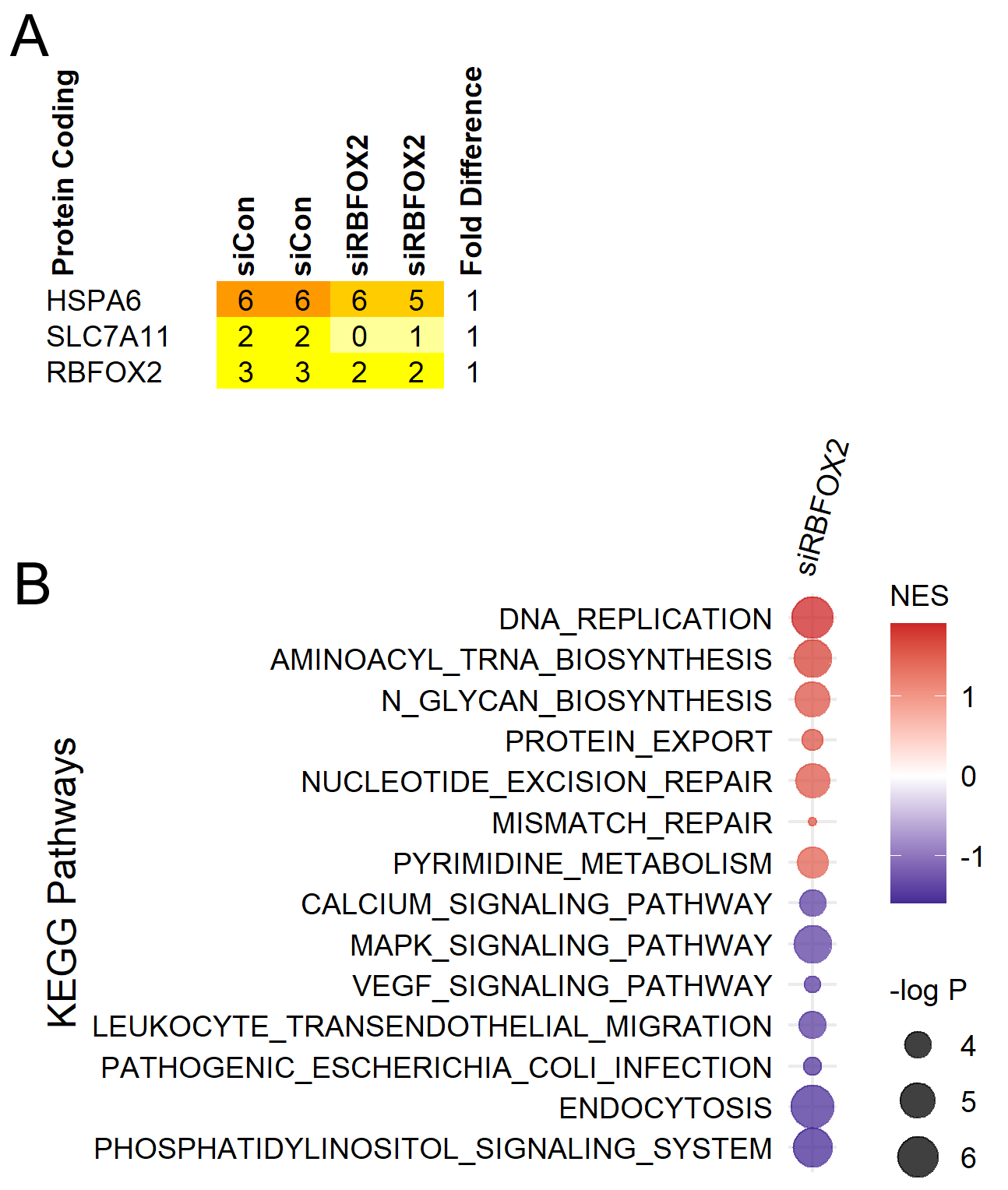

Supplement: Supplementary file 6 [file mmc6.zip › Supplemental Figure 11.tif]

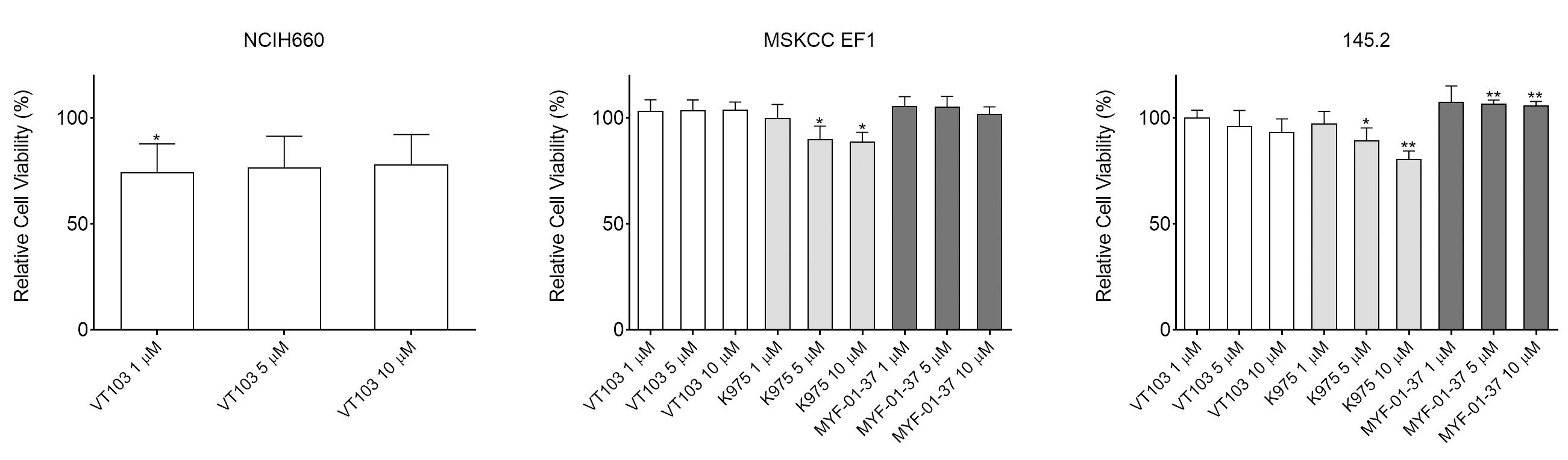

Supplement: Supplementary file 6 [file mmc6.zip › Supplemental Figure 12.tif]

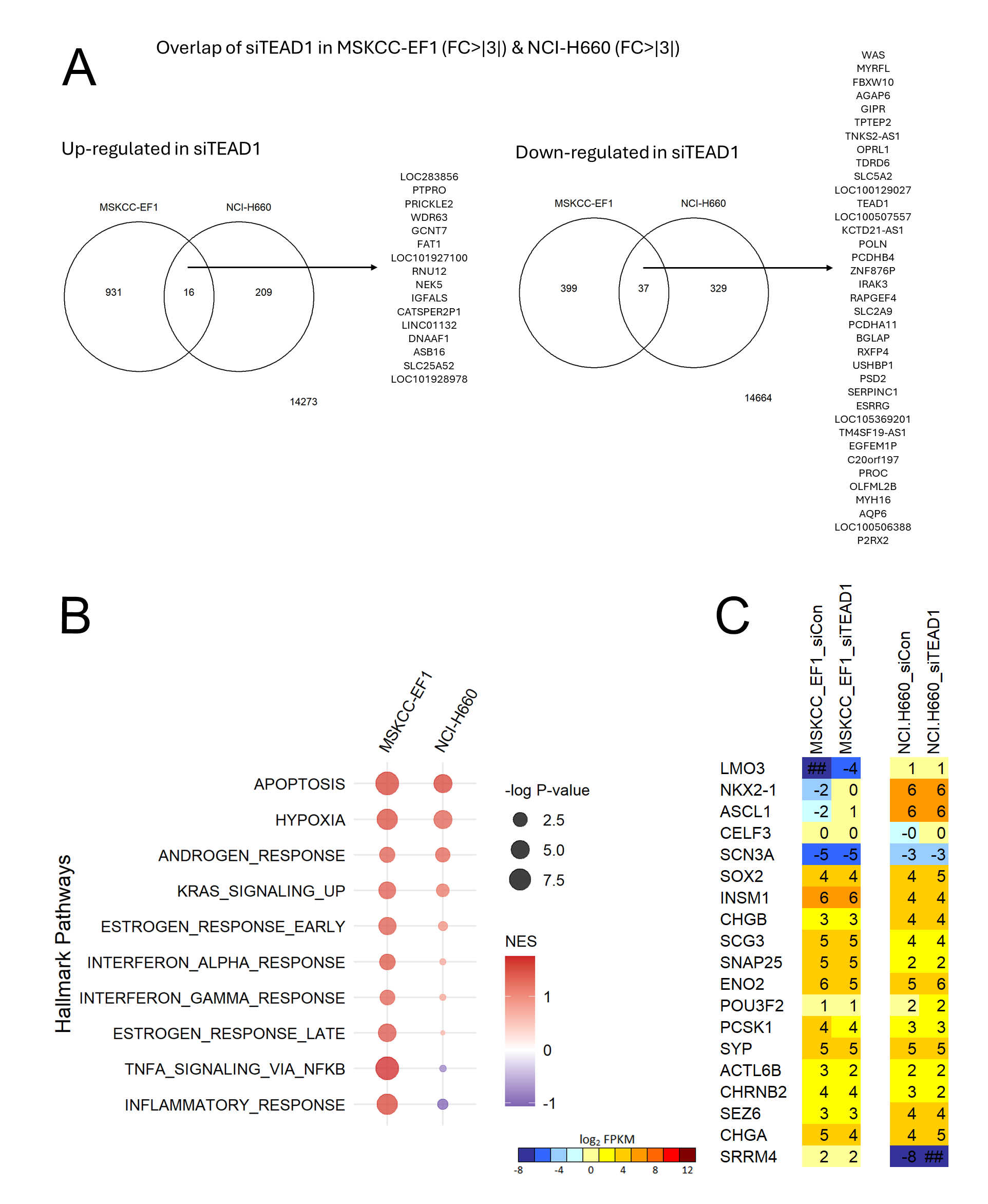

Supplement: Supplementary file 6 [file mmc6.zip › Supplemental Figure 13.tif]
